# Rethinking neurophysiology of delayed onset muscle soreness: updated concepts and mechanisms for mitigation across endurance, resistance, and plyometric exercise

**DOI:** 10.3389/fspor.2026.1709704

**Published:** 2026-09-17

**Authors:** Monèm Jemni, Nicole Quodling, Nadhir Hammami, Shad Groves, Azka Ijaz, Daria Pulyanova, Norman H. Hoffman, Lindsay Bottoms, Frederick R. Carrick, Yaodong Gu

**Affiliations:** 1 Faculty of Sports Science, Ningbo University, Ningbo, China; 2 Carrick Institute, Department of Neuroscience, Cape Canaveral, FL, United States; 3 Research Unit Sport Sciences, Health and Movement (UR22JS01), High Institute of Sport and Physical Education of Kef, University of Jendouba, El Kef, Tunisia; 4 Exercise Biology Laboratory for Performance and Health (LBEPS), University Evry-Paris Saclay, Evry, France; 5 School of Health, Medicine and Life Sciences, University of Hertfordshire, Hatfield, Hertfordshire, United Kingdom; 6 College of Medicine, University of Central Florida, Orlando, FL, United States; 7 Burnett School of Biomedical Science, University of Central Florida, Orlando, FL, United States; 8 MGH Institute for Health Professions, Boston, MA, United States

**Keywords:** delayed onset muscle soreness, eccentric exercise, endurance, exercise modality, neurophysiology, plyometric, recovery strategies, rehabilitation

## Abstract

Delayed-onset muscle soreness (DOMS) is a physiological response to intense eccentric exercise across endurance, resistance, and plyometric training. Although traditionally attributed primarily to structural muscle damage and inflammation, accumulating evidence indicates that DOMS is a multifactorial neurophysiological response involving complex interactions between peripheral tissue responses, inflammatory signalling, neural sensitisation, proprioceptive alterations, and central nervous system adaptations.

**Objective:**

This review aimed to synthesise current evidence on the neurophysiological mechanisms underlying DOMS across endurance, resistance, and plyometric exercise within an integrated neurophysiological framework. It further examined the mechanisms underpinning therapeutic interventions, critically evaluated current approaches to DOMS assessment, and explored their implications for personalised recovery strategies, providing key-take-home to help clinicians and field partitioners.

**Methods:**

This systematic review with narrative synthesis followed PRISMA 2020 guidelines and registered with the International Prospective Register of Systematic Reviews (PROSPERO). 1,183 articles, including clinical trials examining DOMS neurophysiology and therapeutic interventions across exercise modalities were screened.

**Results:**

The evidence indicates that DOMS cannot be explained by a single biological mechanism. Rather, distinct exercise modalities activate overlapping but different combinations of inflammatory, neural, metabolic, and proprioceptive pathways that influence symptom development and recovery. The findings further indicate that different exercise modalities elicit unique neuromuscular adaptations, with endurance training leading to cumulative inflammatory responses, resistance training inducing nociceptor sensitization, and plyometric exercise disrupting proprioceptive control. Endurance exercise produces IL-6 increase within 48 h but shows inflammation reduction after repeated bouts. Resistance training causes strength loss with Group III/IV nociceptor activation. Plyometric exercise disrupts proprioception alongside C-fiber sensitization. Recovery interventions show DOMS reduction: compression garments SMD = −0.44, acupuncture SMD = −0.89, foam rolling SMD = −0.34, but majority of studies lack extractable effect sizes preventing meta-analysis. Intervention efficacy appeared to vary according to exercise modality, exercise load, and individual neurophysiological responses, suggesting a dose-response influence on recovery outcomes.

**Conclusion:**

Collectively, the evidence supports the concept that DOMS should be regarded as a multifactorial neurophysiological response rather than simply a consequence of muscle damage. Exercise-specific recovery optimizes DOMS management: active recovery for endurance, compression/photobiomodulation for resistance, foam rolling/acupuncture for plyometric. Chronic training reduces DOMS through repeated bout adaptation. Future research should prioritise standardised outcome reporting together with mechanistic biomarkers and adequately designed longitudinal studies to strengthen the evidence base. This review highlights the need for individualized and exercise-specific recovery strategies to optimize DOMS mitigation, post-exercise recovery, and requires rehabilitation protocols based on neural and biochemical markers. Recovery interventions should also consider the dose-response relationship between exercise load and neurophysiological stress, as treatment effectiveness may vary according to DOMS severity and training demands. Given the variability in individual responses, integrating multiple assessment tools enhances accuracy and reliability in both research and clinical settings.

**Systematic Review Registration:**

https://www.crd.york.ac.uk/PROSPERO/view/CRD420261294291, PROSPERO CRD420261294291.

## 1 Introduction

Delayed Onset Muscle Soreness (DOMS) is a transient physiological response to unaccustomed or high-intensity exercise, particularly when eccentric muscle contractions are involved. It is characterized by muscle pain, stiffness, swelling, reduced range of motion, impaired proprioception, and temporary loss of strength. Symptoms typically develop within several hours following exercise, peak between 24 and 72 h, and generally resolve within 5–7 days (1–9). DOMS affects individuals across all levels of training status but is most pronounced following novel or mechanically demanding loading patterns such as resistance exercise, plyometric movements, or downhill running (7, 10–15).

Earlier hypotheses attributed DOMS to the accumulation of metabolic by-products such as lactate; however, this explanation has been conclusively refuted. Contemporary evidence supports a multifactorial model in which DOMS emerges from the interaction of inflammatory processes (4, 16, 17), nociceptor sensitization (9, 18), neuromuscular dysfunction (10), and central nervous system involvement (18, 19) rather than from a single structural cause.

Although Delayed Onset Muscle Soreness (DOMS) often co-occurs with Exercise-Induced Muscle Damage (EIMD), growing evidence indicates that they are not interchangeable. Advanced imaging techniques, including high-resolution and ultra-high-field MRI, biochemical markers, and neurophysiological assessments demonstrate that DOMS can occur without detectable microscopic muscle fiber disruption (20–25). These findings highlight the role of neurophysiological mechanisms beyond overt structural damage.

A clear distinction between DOMS and EIMD is therefore essential. EIMD is typically associated with structural disruption and pronounced inflammatory responses, including elevations in creatine kinase (CK) (6, 26, 27), lactate dehydrogenase (LDH) (14), C-reactive protein (CRP) (28), and interleukin-6 (IL-6) (4, 20–23, 29). Additional biomarkers such as alanine aminotransferase (ALT) and aspartate aminotransferase (AST) further reflect tissue damage and systemic inflammation (21). In contrast, DOMS involves complex sensory and neuromuscular processes, including altered afferent feedback, peripheral and central sensitization, and transient impairments in motor control.

Imaging-based classification systems reinforce this distinction. The Peetrons ultrasound grading scale and its MRI adaptations categorize muscle injuries from Grade 0 (no detectable lesion) to Grade III (complete rupture) (24). Within this framework, DOMS is classified as a Grade I injury, characterized primarily by edema without significant architectural disruption (23, 24). Supporting this, 7T MRI of the gastrocnemius following strenuous exercise-induced DOMS revealed no microscopic muscle injury (20), suggesting neural and metabolic contributions rather than direct fiber damage.

Neurophysiological evidence further differentiates DOMS from EIMD. Cabral et al. (25) demonstrated persistent disruptions in M-wave surface EMG following eccentric contractions that induced DOMS, indicating transient neuromuscular dysfunction rather than structural injury. Collectively, these findings underscore the need to distinguish DOMS from EIMD, as they arise from distinct mechanisms and warrant tailored clinical and preventive strategies.

Endurance, resistance, and plyometric exercises represent physiologically and motorically distinct training modalities. Endurance exercise is characterized by prolonged, repetitive, predominantly submaximal muscular activity with a substantial metabolic demand and cumulative eccentric loading, particularly during downhill running or deceleration phases (30–32). Resistance exercise involves high-load or high-tension contractions against external resistance, frequently incorporating eccentric actions that impose significant mechanical stress on muscle fibers and connective tissue (6, 10, 12, 15). In contrast, plyometric exercise is defined by rapid stretch–shortening cycle movements, combining high strain rates and explosive eccentric–concentric transitions that place considerable demands on neuromuscular coordination and sensory feedback mechanisms (4, 13, 14, 33).

The magnitude of DOMS is not solely determined by the presence of eccentric contractions but also by factors such as exercise intensity, duration, volume, muscle mass involved, and the individual's training status. Consequently, similar eccentric loading patterns may produce markedly different soreness responses between trained and untrained individuals or between endurance, resistance, and plyometric activities. Furthermore, the assessment of DOMS requires a combination of subjective and objective methodologies. Perceived soreness is commonly quantified using validated pain scales such as the Visual Analogue Scale (VAS) and numerical rating scales, whereas pressure pain threshold, muscle function tests, biochemical markers, and imaging techniques provide complementary physiological information (9, 34). This multidimensional approach is increasingly recommended because pain perception alone may not accurately reflect the extent of neuromuscular dysfunction, tissue stress, or recovery status (9, 35).

Despite their physiological and mechanical differences, these exercise modalities share common neurophysiological features relevant to the development of DOMS. All involve eccentric loading to varying degrees, provoke inflammatory signaling cascades, and induce peripheral and central sensitization of nociceptive pathways. However, the relative contribution of mechanical stress, metabolic load, sensory afferent activation, and central processing differs across modalities. This variability suggests that DOMS cannot be fully explained by a uniform structural damage model and supports the need for an integrative, modality-specific analysis grounded in neurophysiology.

The impact of DOMS extends beyond transient discomfort, influencing neuromuscular coordination, proprioception, and movement efficiency. Altered afferent feedback and motor control during DOMS may increase injury risk and negatively affect both athletic performance and rehabilitation outcomes (8, 36). Similar relationships between pain, motor control dysfunction, and impaired movement quality have been reported in chronic pain conditions characterized by central sensitization (26). Consequently, a deeper understanding of the neurophysiological mechanisms underlying DOMS across different exercise modalities is essential for the development of effective, evidence-based recovery strategies.

The impact of DOMS extends beyond transient discomfort, influencing neuromuscular coordination, proprioception, and movement efficiency. Altered afferent feedback and motor control during DOMS may increase injury risk and negatively affect both athletic performance and rehabilitation outcomes.

### 1.1 Research gap

While prior reviews document DOMS interventions (compression 23, 46, 69, 72, acupuncture 91, foam rolling 79) and exercise-induced muscle damage mechanisms, they fail to integrate neural mechanisms with modality-specific pathophysiology. Existing literature treats endurance (26, 29–36), resistance (6, 10), and plyometric DOMS as mechanistically equivalent, overlooking:

- Endurance repeated bout adaptation (31) vs. resistance inflammatory response (37)
- Nociceptor profiles: metabolic activation (30) vs. mechanical Group III/IV sensitization (9, 18)
- Central processing: periaqueductal gray (PAG)-rostral ventromedial medulla (RVM) axis inhibition (PAG-RVM) (19)
- Adaptation kinetics: endurance vs. structural satellite cell repair (10)

This review addresses three critical research gaps: (1) modality-specific neural pathways, (2) integration of peripheral nociception with central sensitization, and (3) chronological adaptation from acute inflammation to chronic protection. No prior synthesis links all 9 studies that report effect sizes (17, 28, 38–44) with primary neurophysiology (9, 18, 19, 27, 31) across the acute-subacute-chronic continuum.

Collectively, the evidence reviewed in this manuscript supports the concept that delayed-onset muscle soreness (DOMS) should be viewed as a multifactorial neurophysiological response rather than simply a consequence of structural muscle damage. Accordingly, this review adopts an integrated neurophysiological perspective that brings together current evidence on peripheral tissue responses, inflammatory signalling, neural sensitisation, proprioceptive alterations, and central nervous system adaptations. Figure 1 presents the conceptual model illustrating the multifactorial neurophysiological mechanisms underlying delayed-onset muscle soreness (DOMS). Unaccustomed mechanical loading initiates structural and connective tissue stress, inflammatory signalling, peripheral nociceptor sensitisation, and central nervous system adaptations, which collectively contribute to pain perception, proprioceptive alterations, motor adaptations, and functional recovery. Therapeutic interventions may influence one or several stages of this pathway.

**Figure 1:**
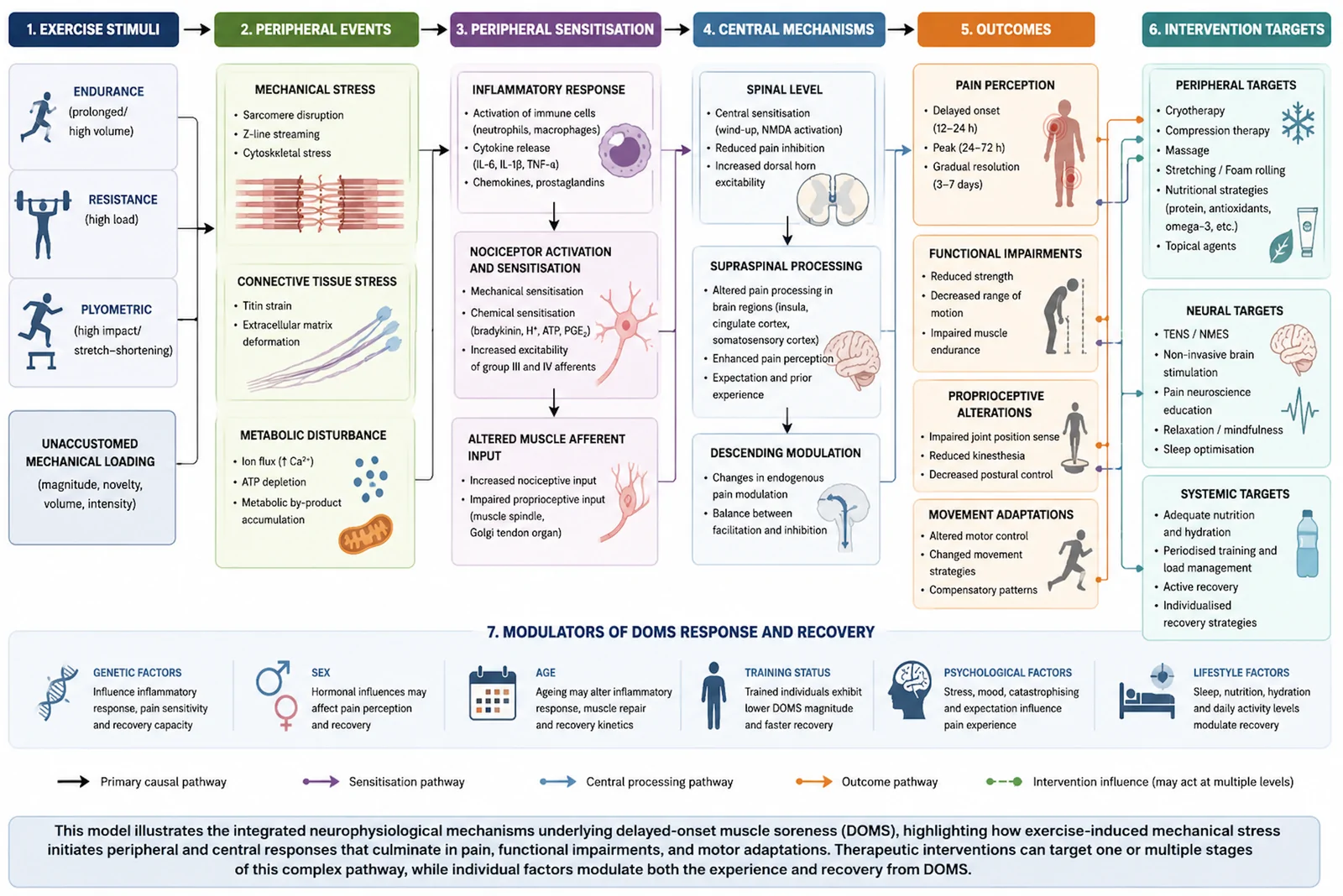
Integrated neurophysiological mechanisms underpinning DOMS.

Accordingly, the primary objective of this review is to synthesise current evidence on the neurophysiological mechanisms underlying DOMS following endurance, resistance, and plyometric exercise. By comparing modality-specific pathways of nociception, neuromuscular adaptation, inflammatory responses, proprioceptive alterations, and central sensitisation, the review seeks to explain why DOMS manifests differently across exercise modalities and why commonly used recovery interventions demonstrate variable efficacy. A secondary objective is to critically examine the methods currently used to assess DOMS and their respective strengths and limitations. Ultimately, by adopting this neuroscience-informed framework, the review aims to support more personalised, exercise-specific approaches to DOMS assessment, management, and post-exercise recovery.

## 2 Method

This systematic review with narrative synthesis was conducted in accordance with PRISMA 2020 guidelines (Item 4) to ensure transparency and reproducibility. It was prospectively registered with the International Prospective Register of Systematic Reviews (PROSPERO; registration number: CRD420261294291, 2026). Systematic procedures included predefined PICOS criteria, a comprehensive search yielding 1,183 records, dual independent reviewer screening (Figure 2), and methodological quality assessment (Table 3). Narrative synthesis was selected due to the absence of extractable effect sizes in the majority of included studies, precluding meta-analysis (PRISMA Item 21b).

**Figure 2:**
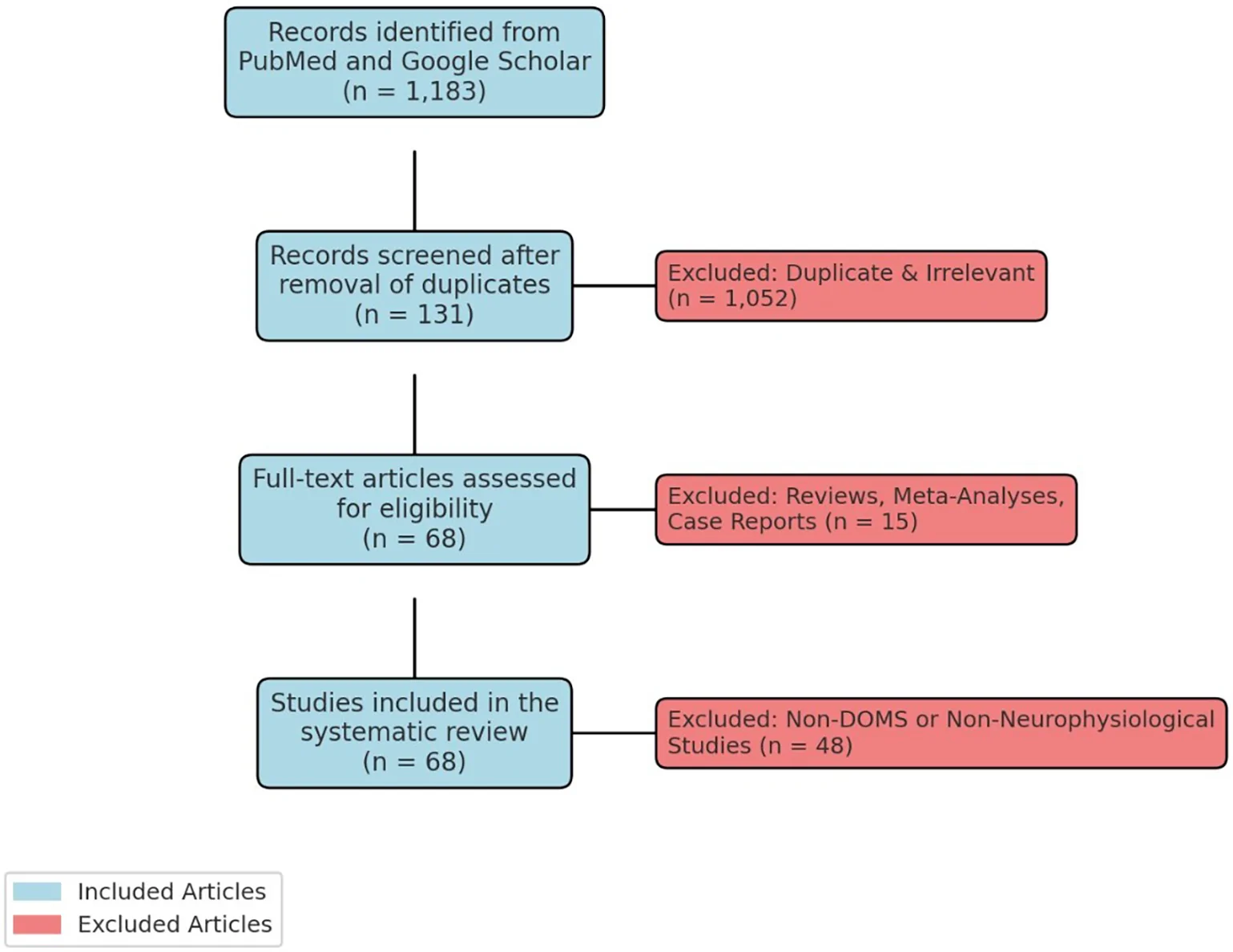
PRISMA 2020 flow diagram of studies’ selection.

The review focused on neurophysiological mechanisms underpinning Delayed Onset Muscle Soreness (DOMS) and recovery mechanisms across endurance, resistance, and plyometric training modalities.

To contextualize the research, the following Population, Interventions, Comparators and Outcomes (PICOS) framework was applied:

- Research Question 1: What are the contemporary neurophysiological mechanisms underpinning DOMS severity across endurance, resistance, and plyometric exercise modalities?
- Research Question 2: Among healthy adults experiencing exercise-induced DOMS, what is the effect of neurophysiologically targeted recovery interventions compared to standard care on DOMS severity, strength recovery, and biochemical markers?
- Study Designs: RCTs, controlled trials, prospective cohort studies (2014–2024)
- Population: Healthy adults experiencing DOMS after endurance, resistance, or plyometric exercise
- Interventions: Recovery strategies targeting neurophysiological mechanisms (e.g., nociceptor desensitization, inflammation modulation, central inhibition restoration)
- Comparators: Passive recovery, placebo, no intervention, or alternative recovery modalities
- Outcomes:
  - Primary: DOMS severity (VAS), Pressure Pain Threshold (PPT)
  - Secondary: Strength loss (MVC), biochemical markers (CK, IL-6), neurophysiological measures (EMG, H-reflex)

### 2.1 Search strategy

A systematic literature search was conducted using PubMed (primary database) and Google Scholar (secondary database) to identify relevant studies published between January 1, 2014, and March 25, 2024. The search was finalized on March 25, 2024. A 10-year timeframe was selected to ensure inclusion of contemporary mechanistic and intervention-based evidence.

Search terms included the following Boolean combinations:

- “DOMS” OR “Delayed Onset Muscle Soreness” AND
- “Neurophysiology” OR “Neuro-physiology” AND
- “Fatigue” AND
- “Exercise-Induced Muscle Injury” AND
- “Imaging”

Filters were applied for human studies, English language, peer-reviewed publications, and full-text availability.

No systematic review management software was used. All records were exported and managed manually using a structured open-access spreadsheet that has been transformed into Table 1.

**Table 1:** Neuro-physiological mechanisms underpinning exercise-related DOMS to endurance, resistance, and plyometric, and summary of their treatment effectiveness.

Ex type	Author and year	Participants (age, sex, number)	4.Measures	Treatment	Findings/Effect on DOMS	Neuro-physiological explanation
Endurance	Close et al. (30)	24 males. Age not specified	Ascorbate Malonaldehyde total glutathione isokinetic dynamometry	The ascorbic acid group received 1 g ascorbic acid 2 h pre-exercise	Ascorbic acid supplementation attenuates ROS production following downhill running, without affecting DOMS. Furthermore, ascorbic acid supplementation may inhibit the recovery of muscle function.	Muscle damage and increase in ROS production.
	Etheridge et al. (45)	9 active recreational males	Quadriceps maximum isometric voluntary contraction (MVC).	30 min downhill run at a −10° gradient, targeting 75% of age-predicted heart rate maximum.	Both groups showed significant increases in CK, PC, and perceived muscle soreness at 24 h post-exercise.	The ingestion of a protein-rich supplement immediately after eccentric exercise may facilitate muscle repair and reduce muscle soreness by providing essential amino acids necessary for muscle protein synthesis.
			Peak 5 s power output (PPO).	Immediately post-exercise, participants ingested either:	In the CON group, MVC declined by 7.9% at 24 h and reached a 10% reduction at 48 h.	These findings suggest that a single protein meal post-exercise can enhance the recovery of muscle function following eccentric exercise-induced muscle damage.
			Perceived muscle soreness.	- 100 g of protein solution containing 40 grams of essential amino acids (PRO).	In the PRO group, MVC remained at pre-exercise levels throughout the 72 h recovery period.	
			Plasma creatine kinase (CK) levels.	- Placebo solution (CON).	PPO showed a similar trend, with an 8.7% decline at 48 h in the CON group, while the PRO group maintained pre-exercise values.	
			Protein carbonyl (PC) content			
	Jafariyan et al. (31)	11 females. Age 20–30 years old	TLC	None	Pro- and anti-inflammatory cytokine responses and changes in leukocyte counts following the second and third bouts tended to moderate inflammatory responses and return to baseline levels. In addition, there was moderate muscle soreness following the 3-day downhill running, concomitant with strength recovery on the third day. Moreover, a continuous increase in serum levels of intramuscular proteins implied that the clearance rate increased from tissue to circulation. Finally, early adaptation to exercise could be due to the repeated bout effect.	Upon irritant infliction, local macrophages and other cells sense the insult and produce a panel of inflammatory mediators, such as cytokines and chemokines. These mediators stimulate the nearby microvasculature and attract large numbers of leukocytes to migrate across the vascular wall and infiltrate into tissues.
			DLC			
			CK			
			LDH			
			Mb			
			IL-10, IL-6			
			MCP-1			
			Goniometer ROM			
	Marquet et al. (32)	7 males and 4 females. Average age 20 years old	Maximal sprint power output test	Nutritional strategy	A nutritional strategy immediately after exercise appears to be the most effective recovery strategy due to its positive impact on performance, perception of DOMS, and fatigue. Cold water immersion is a complementary strategy to decrease acute fatigue between training sessions.	Cold immersion decreases post-exercise inflammation, simultaneously reducing delayed-onset muscle soreness (DOMS). Cold-induced vasoconstriction also accelerates the reactivation of the parasympathetic nervous system immediately after exercise. Nutrition is one of the most effective ways to achieve complete recovery, as high-intensity exercise leads to both the depletion of muscular glycogen and an increase in the rate of muscle protein degradation.
				Cold water immersion		
	Nosaka et al. (38)	16 males. Age 20–24 years old	Muscle soreness.	- Amino acid supplementation	- No significant differences in muscle soreness or damage indicators between amino acid and placebo conditions in Experiment 1	- Amino acid supplementation may attenuate DOMS and muscle damage when ingested during recovery days.
			Plasma levels of creatine kinase (CK), aldolase, and myoglobin as indicators of muscle damage.	- Two experimental protocols:	- In Experiment 2, amino acid supplementation resulted in significantly lower plasma CK, aldolase, myoglobin levels, and reduced muscle soreness compared to the placebo.	Timing and frequency of amino acid supplementation are crucial factors in mitigating exercise-induced muscle soreness and damage.
				*Experiment 1: Supplement ingested 30 min before and immediately after exercise.		
				*Experiment 2: Supplement ingested 30 min before, immediately after, and 8 additional times over four days post-exercise.		
	Ouergui et al. (29)	61 judo athletes (35 males and 26 female). Age 14–17 years old	- Total Quality of Recovery (TQR).	- 3 experimental groups (randori, uchi-komi, and running) and a control group (regular training).	Significant correlations between well-being indices and recovery state during both periods.	- Intense training, especially eccentric exercises (e.g., randori), causes micro-tears in muscle fibers, leading to DOMS. The high-intensity design likely contributed to this damage.
			- Well-Being Indices (Sleep quality, Stress levels, Fatigue levels, DOMS, Hooper Index (HI)).	- Training protocol: Intensified Training Phase (4 sessions/week for 4 weeks, incorporating high-intensity interval training (HIIT) specific to judo), and Tapering Phase (Followed the intensified training for 12 days).	In the intensified training phase, TQR was negatively correlated with DOMS, indicating that higher muscle soreness was associated with poorer recovery. Session-RPE was positively correlated with DOMS, suggesting that higher perceived exertion was linked to increased muscle soreness.	- Muscle damage triggers an inflammatory response, releasing mediators that heighten soreness, reflected in well-being indices.
			- Session-RPE (recorded 15–30 minutes after each training session).	- Training session duration: 1h30 including a standardized warm-up.	During the tapering phase, TQR showed a negative correlation with DOMS reinforcing the idea that effective recovery strategies can mitigate muscle soreness.	- TQR and well-being indices (sleep, fatigue) gauge recovery. Poor recovery prolongs DOMS by impairing muscle repair.
			- Physical Enjoyment by the Physical Activity Enjoyment Scale (PACES) after intensified and tapering training periods.			- Stress and fatigue can amplify soreness perception, while good sleep and recovery improve muscle repair.
Resistance	Aminian-Far et al. (6)	22 females and 10 males. Average age 21 years old	PPT	The whole-body vibration-training group (*n* 15) was exposed to a vibration treatment consisting of one 60 s bout at 35 Hz transmitted through a vibration platform before performing an exercise protocol	Administered before eccentric exercise, WBVT may reduce DOMS, attenuating PPT and plasma CK activity, and controlling strength loss after a bout of eccentric exercise.	Increased serum CK activity, ultrastructural disruption, inflammation, increased proteolytic activity, Disruption of the normal banding patterns of skeletal muscle and broadening or complete disruption of sarcomere Z lines. Edema and sensitisation of afferent fibres resulting from the production of prostaglandin E_2._
			CK			
			Strength			
	Antonialli et al. (39)	40 males healthy untrained. Age 18–35 years old	- MVC (isokinetic dynamometer, at various time points (pre-exercise, immediately post-exercise, and at 1, 24, 48, 72, and 96 h post-exercise)).	- A randomized, double-blinded, placebo-controlled trial	Phototherapy significantly improved MVC and reduced DOMS compared to the placebo group. DOMS was significantly decreased with the 30 J dose from 24 to 96 h post-exercise and with the 50 J dose from immediately after to 96 h post-exercise.	- Phototherapy enhances blood flow to irradiated tissues, facilitating the removal of metabolic waste and improving nutrient delivery for muscle recovery.
			- DOMS (with VAS and Algometry; pain threshold in the knee extensors).	- 4 groups receiving different doses of phototherapy (10 J, 30 J, 50 J, or placebo) applied to six quadriceps sites before an eccentric exercise protocol of 75 contractions.		- Low-level laser therapy (LLLT) stimulates mitochondrial function, increasing ATP production, which is vital for muscle repair and performance.
			- CK Activity	- The phototherapy combined super-pulsed laser and light-emitting diodes (LEDs) at specific wavelengths (905, 875, and 670 nm).		- LLLT also reduces reactive oxygen species (ROS) and boosts antioxidant activity, protecting muscle tissues from exercise-induced damage and inflammation.
						- The reduction in DOMS may stem from altered pain pathways; by increasing pain thresholds (measured by algometry), phototherapy may change pain perception through neurophysiological mechanisms, potentially involving endorphin release.
	Aver Vanin et al. (40)	28 high-level male soccer players. Age 18–35 years old	- MVC (isokinetic dynamometer).	- A randomized, double-blind, placebo-controlled clinical trial was performed.	- LLLT significantly increased MVC at 50 J dose immediately after exercise and 10 J dose from 24 to 96 h post-exercise.	- LLLT enhances mitochondrial function, so improve muscle performance and recovery.
			- DOMS (analog algometer and VAS for pain intensity).	- 4 groups (3 active treatment doses and one placebo).	- Both 10 J and 50 J doses significantly decreased CK activity and IL-6 levels compared to placebo	- The decrease in IL-6 levels suggests that LLLT may help modulate inflammation, potentially reducing muscle damage markers like CK.
			- CK Activity	- The treatment involved pre-exercise application of low-level laser therapy (LLLT) at three different doses (10 J, 30 J, and 50 J) using a cluster laser device emitting at 810 nm, to 6 sites on the quadriceps muscle 2 min before performing a 75 eccentric exercise protocol contractions.	- LLLT did not show a significant effect on reducing DOMS, as measured by both algometry and VAS, across all treatment groups.	- LLLT's lack of effect on DOMS suggests that pain perception and muscle soreness involve complex mechanisms beyond inflammation. This includes both peripheral and central sensitization of nociceptive pathways, which may not be fully influenced by LLLT.
			- Interleukin-6 (IL-6) Expression using the ELISA method.	- Assessment Timing: before exercise, immediately after, and at intervals of 1, 24, 48, 72, and 96 h post-exercise.		
	Bourgeois et al. (41)	8 males. Age 20–27 years old	CK	Subjects were randomised to ingest 500 mg of enteric coated naproxen sodium or an identical placebo 4 h before exercise protocol and every 12 h postexercise, for 48 h postexercise.	Naproxen does not alter indices of muscle damage in resistance-exercise trained men.	Disrupted muscle fibres, Edema, increased CK, and inflammation are mediated at least partially by the release of prostaglandins. Oxygen free radicals may also be implicated in damaging muscle membranes.
			Inflammatory cell			
			Muscle force			
	Connolly et al. (3)	13 females and 11 males. Age not specified	Strength of the elbows flexors	1,000 mg of ascorbic acid 3 times daily	Vit C protocol of 3 × 1,000 mg/day for 8 days is ineffective in protecting against selected markers of DOMS.	Increased muscle pressure, prostaglandin production, calcium loss within muscle cells, and free radical production.
	Crenshaw et al. (10)	8 males. Age 21–40 years old	Peak intramuscular pressure and torque	None	DOMS was experienced exclusively for the leg eccentric activity.	Structural muscle damage caused by high tension associated with eccentric activity
			Comparison of concentric vs. eccentric activity			
	Cristina-Souza et al. (42)	10 young male athletes (runners, sprinters, jumpers). Age 13–17 years old	- Electromyographic (EMG) Activity (vastus lateralis during half-squat exercises). - Rating of Perceived Exertion (RPE) - DOMS (visual analog scale) - Plasma Lactate-Dehydrogenase (LDH) - Creatine Kinase (CK) - MVIC	A randomized, double blind, crossover design. Consumption either Panax ginseng (100 mg·kg^−1^·d^−1^) or a placebo (starch) for 8 days. Three doses: at breakfast, 1 h before training, and before bedtime. 7 days washout period between the two experimental was implemented.	- Attenuated Perceived Effort (RPE scores were significantly lower in the ginseng condition). - Accelerated Recovery of Muscle Force (MVIC returned to baseline levels 24 h post-exercise with ginseng, remained reduced for 48 h in the placebo group). - No Significant Effect on DOMS between the ginseng and placebo groups, indicating that while ginseng improved recovery of muscle force, it did not significantly affect muscle soreness.	The supplementation likely enhanced neuromuscular function, possibly through the modulation of neurotransmitters and increased catecholamine levels, which facilitate muscle activation. The increased muscle excitation may have alleviated stress on individual muscle fibers, leading to fewer afferent signals sent to the central nervous system, thereby reducing the perception of effort during exercise. The MVIC accelerated recovery suggests that Panax ginseng may improve neuromuscular recovery post-exercise, potentially through mechanisms that enhance calcium channel activation and sodium-potassium pump interactions, crucial for muscle contraction and recovery
	Croisier et al. (22)	10 males. Average age 24 years old	Serum CK myoglobin	None	Maximal isometric, eccentric contractions create muscle damage as measured by VAS, serum CK and myoglobin.	DOMs are associated with an inflammatory reaction and increased CK, myoglobulin, and PMN.
	Curtis et al. (5)	18 males and 17 females. Age 20–40 years old	VAS only	20 min Frequency specific microcurrent (FSM) programme to one leg, randomly chosen	FSM therapy provided significant protection from post-exercise muscle soreness.	DOMS is attributed to an accumulation of the metabolic end products of exercise, resulting in elevated muscle lactate. This assumption is now understood to be unconnected to DOMS. It is now proposed that the soreness may be the result of mechanical or biochemical factors
	Da Silva et al. (7)	20 males. Average age 24 years old	CK	green tea extract for 15 days with a dosage of 500 mg/day	Green tea extract supplementation can be a strategy to reduce muscle damage during exercise recovery. However, muscle soreness, oxidative damage, and antioxidant status remained unaltered in response to supplementation.	Among the mechanisms of DOMS is the mechanical stimulus during exercise acting as a trigger of an inflammation condition generating reactive species of oxygen (ROS) that damages muscle structure due to impaired antioxidant capacity
	Dierking et al. (43)	40 females. Age 18–40 years old	Spontaneous muscle shortening was assessed with a goniometer. Diagnostic ultrasound.	None	Diagnostic US assessment of muscle soreness, as evidenced by intramuscular swelling, was limited, and the technique lacked sensitivity to detect any statistically significant changes in muscle.	Eccentric contraction overstretches the sarcomeres, which allows excessive calcium to enter the muscle fibres from the surrounding interstitial fluid. This calcium influx might then activate a protease system, which would digest structural proteins and cause muscle damage and changes in membrane permeability.
	De Oliveira et al. (16)	28 high-level male soccer players. Age 18–35 years old	- MVC (isokinetic dynamometer). - DOMS (analog algometer and VAS for pain intensity). - Biochemical Markers (CK and lactate dehydrogenase), inflammation (IL-1b, IL-6, TNF-a), and oxidative stress (catalase, superoxide dismutase, carbonylated proteins, and thiobarbituric acid).	- A randomized, double-blind, placebo-controlled clinical trial was performed. - 4 groups: 3 active treatment of Photobiomodulation Therapy (PBMT) (outputs: 100, 200, 400 mW) and one placebo (0 mW). - PBMT was administered before an eccentric contraction protocol using a five-diode cluster laser device with a wavelength of 810 nm, delivering a dose of 10 J per diode. - The treatment was applied to six sites on the knee extensors, with exposure times varying according to the power output. - Evaluations were conducted before exercise and at multiple time points post-exercise (1 min, 1, 24, 48, 72, and 96 h).	PBMT significantly increased MIVC and decreased DOMS across all power output groups compared to the placebo. Notably, the group receiving 100 mW showed the most substantial reduction in DOMS, with significant differences observed immediately after exercise and at 1, 24, and 48 h post-exercise. The 100 mW group maintained a higher-pressure pain threshold compared to the placebo group.	PBMT boosts circulation in the treated area, aiding in the removal of muscle damage waste. The therapy reduces inflammatory markers (IL-6, TNF-a) post-exercise, leading to less pain and better recovery. PBMT lowers oxidative stress markers, indicating less damage to muscle tissues.
	Dupuy et al. (17)	1,188 gender not specified. Age 18–65 years old	Meta-analysis evaluating the impact of recovery techniques on DOMS, perceived fatigue, muscle damage, and inflammatory markers after physical exercise	Massage Compression garments Water immersion	Massage is the most effective method for reducing DOMS and perceived fatigue. Perceived fatigue can be managed using compression garments, massage, or water immersion.	Changes in the blood concentrations of muscle damage indicators [i.e., creatine kinase (CK)] and inflammatory biomarkers [C-reactive protein (CRP) and interleukin-6 (IL-6)] that are observed after exercise and are associated with the occurrence of DOMs
	Goulart et al. (44)	14 resistance-trained males. Age 24 ± 2 years old	- Volume Load (VL). - First Set Volume Load (FSVL) - CMJ - MVIC - CK - DOMS (using a 7-point Likert scale).	Resistance training (RT) protocol. 3 experimental conditions: with varying recovery intervals (24, 48, and 72 h). Each condition included 2 training sessions: 5 × 8-10 until concentric failure (squat, leg press exercises)	Significant changes in performance and perceptual responses over the recovery intervals A recovery interval of at least 48 h was necessary for the reestablishment of performance variables, while 72 h provided better perceptual responses	The increase in CK levels at 24 and 48 h after training suggests muscle damage associated with the inflammatory response required for muscle repair that can lead to DOMS characterized by muscle soreness and stiffness. Despite the presence of soreness, participants were able to maintain their performance at 48 h, indicating that the neuromuscular system can adapt to pain and continue to function effectively. The significant correlation between viral load and performance measures (FSVL, CMJ, MVIC) suggests that the ability of the neuromuscular system to recover and function may be influenced by the extent of muscle damage and expected recovery intervals.
	Hübscher et al. (12)	10 males and 12 females Age 22–30 years old	Mechanical pain threshold (MPT) maximum isometric voluntary force (MIVF)	Acupuncture	Acupuncture proved to reduce perceived pain arising from exercise-induced muscle soreness.	High muscle tension developed during eccentric contractions may precipitate mechanical disruption of myofibrils and connective tissue, which triggers an inflammatory response.
	Kerr et al. (28)	30 males. Age 30–45 years old	Strength Myokines	NPN_1 supplementation	NPN_1 is a characterised plant-based efficacious ingredient that, at a low dose, we have shown to improve strength recovery and reduce fatigue following strenuous activity.	Proposed mechanisms of DOMS include inflammation, calcium channel leakage, oxidative stress and muscle damage
	Kraemer et al. (46)	20 females. Age not specified	Ease of performing daily activities arm girth, resting elbow angle elbow flexor strength blood samples for hormone and enzyme analyses.	Compressive sleeve during recovery period	Compression was found to promote faster recovery of force production, prevent swelling and loss of elbow extension at rest in the exercised arm, and allow subjects to return to daily activities sooner. Subjects treated with the compressive sleeve reported less pain at rest and during palpation.	Eccentric muscle damage results from mechanical stress, muscle cell calcium homeostasis imbalances, or local inflammation.
	Kristensen et al. (9)	28 gender not specified. Age 18–45 years old	PPT isometric force measurement	None	The baseline reduced mean pain tolerance, increased positive affect, facilitated TSP, younger age, completed more exercise repetitions, and predicted muscle pain intensity on Day 2.	Sleep, physical activity, affect and pain-related catastrophizing seem to play an important role in pain development and/or exacerbation
	Machado et al. (47)	30 sedentary males. Age 18–35 years old	- Maximum Voluntary Contraction (MVC) - CK - Blood Lactate Levels - DOMS using a VAS at various time points.	A randomized, triple-blind, placebo-controlled trial. 3 groups: - Placebo (PG): received placebo photobiomodulation therapy combined with static magnetic field (PBMT-sMF) treatment bilaterally to the anterior thigh muscles. - Local Group (LG): received active PBMT-sMF treatment to the anterior thigh muscles of the exercised limb and placebo treatment to the non-exercised limb. - Non-local Group (NLG): received placebo treatment to the exercised limb and active PBMT-sMF treatment to the non-exercised limb. - Active PBMT-sMF was applied using a device with laser diodes and LEDs, delivering a total dose of 180J per thigh.	- Improvements in MVC, lower CK activity, reduced blood lactate levels, and decreased DOMS in LG compared to PG and NLG. - Less muscle soreness at 1, 24, 48, and 72 h post-exercise, indicating effective recovery in LG. - PBMT-sMF applied to non-exercised muscles did not yield beneficial effects in NLG and PG.	- PBMT interacts with cytochrome c-oxidase in mitochondria, enhancing ATP production, which is crucial for muscle function and recovery. The therapy seems t improve local blood flow, facilitating the removal of metabolic waste products (like lactate) and enhancing nutrient delivery to the muscles. - PBMT-sMF may help mitigate muscle damage and soreness post-exercise by modulating inflammatory responses. This may influence pain pathways, leading to a reduction in the perception of soreness and discomfort associated with muscle fatigue.
	Mekjavic et al. (48)	24 males. Age 20–35 years old	Maximum voluntary contraction arm circumference	Hyperbaric environment for 60 min	Hyperbaric oxygen therapy does not enhance recovery of exercise-induced loss of muscle strength nor the recovery from delayed onset muscle soreness.	Signified by morphological changes and elevations of muscle proteins, muscle stiffness, decrements in force production, and edema. Concomitant with the several proposed theories regarding the development of DOMS are numerous suggestions for the treatment of DOMS, including anti-inflammatory drugs, massage, and light exercise.
	Palma et al. (49)	35 active men. Age 18–30 years old	- DOMS using a Numerical Pain Rating Scale (NPRS) 24 h after fatigue induction. - CK levels, pre and post fatigue protocol. - Muscle Strength, Isometric torque. - Functional Performance, horizontal jump tests. - Perceived Exertion immediately after the fatigue protocol.	- A double-blind, randomized controlled study. - 2 groups: G1 (received active photobiomodulation (PBM) 5 min before an eccentric fatigue protocol) and G2 (received active PBM immediately after the fatigue protocol). G1 and G2 also had a placebo treatment. - Fatigue Protocol (eccentric knee extensions on non-dominant leg at 80% of 1RM, 5 × 10). - PBM applied with 13 diodes device, focusing on six points in the quadriceps muscle. The light was delivered for 73 s per point with a total dose of 181.2 J for each session. - A washout period of at least one week.	- Active PBM significantly reduced DOMS compared to placebo, but the clinical impact was minimal (less than 2 points on NPRS). - PBM applied before eccentric fatigue did not significantly reduce DOMS vs. placebo. It led to a smaller CK increase, suggesting a protective effect against muscle damage.	PBM modulates inflammation, lowering markers linked to muscle damage and soreness, enhances mitochondrial function, aiding muscle recovery and pain reduction, helps maintain calcium balance, preventing muscle breakdown and soreness, and triggers the release of endorphins, reducing pain perception and muscle soreness.
	Pearcey et al. (8)	8 males. Average age 22 years old	PPT sprint speed (30-m sprint time) power (broad-jump distance) change-of-direction speed (*T*-test) dynamic strength-endurance	foam rolling	Three 20-minute bouts (60 min total) of foam rolling can substantially enhance recovery after DOMS and alleviate muscle tenderness.	Excitation-contraction coupling impairment, damage to various muscle fibers, metabolic impairments, and fatigue have been proposed to explain how DOMS impairs muscle function.
	Pesenti et al. (50)	28 male Soccer players. Age16–19 years old	- Pain Intensity using a numerical scale of pain (NSP) - EMG (Root Mean Square (RMS) values of the vastus medialis oblique (VMO), vastus lateralis (VL), and rectus femoris (RF)) during a kicking task. - Postural Control measurements (center of pressure (COP) oscillation)	Randomized, blinded clinical trial study 4 groups (*n* = 7/group): - Cold Water Immersion group (CWIG) in water at 10 °C - Thermoneutral Water Immersion group (TWIG) in water at room temperature. - Active Recovery group (ARG), 10 min walking on a treadmill - Rest/Control group (CG), 10 min seated and relaxed. - NSP assessed at baseline and at 24, 48, and 72 h post-intervention.	- Pain intensity in the CWIG group returned to baseline levels after 72 h, indicating effective recovery from DOMS. - TWIG, ARG, and CG groups continued to experience higher pain levels at 72 h post-intervention. - DOMS protocol was effective, with pain intensity peaking at 24 and 48 h for all groups, but only the CWIG group returned to baseline pain levels. - Postural control is similar among the groups at any time point.	Cold water immersion (CWI) reduces inflammation by decreasing muscle temperature and metabolic activity, leading to reduced muscle damage post-exercise. It may cause vasoconstriction, which reduces blood flow to the muscles, followed by vasodilation upon rewarming, promoting better circulation and removal of metabolic waste products. It can activate pain receptors and alter pain perception; potentially leading to a temporary reduction in the sensation of pain. It may help maintain muscle function and performance, as indicated by the return to baseline pain levels in the CWIG group.
	Christmas et al. (51)	20 males. Age 18–39 years old	VAS only	None	Performing moderate intensity exercise MIE 48 h following muscle-damaging exercise impairs specific, but not all, physiological outcome variables. On average, perceived readiness decreased by 18% in the experimental group.	Not specified
	Sakamoto et al. (13)	7 males and 5 females. Average age 25 years old	limb circumference static joint angles CK isometric strength dynamic muscle performance involving a stretch-shortening cycle (SSC)	None	Eccentric damage impairs dynamic performance by affecting the muscle itself, the level of motor command via exaggerated pain, and the enhancing effects of the stretch-shortening cycle. The repeated bout effect was present but slightly reduced when subsequent exercise performed in the early recovery phase was intense and differed in mode from the initial damage-inducing eccentric exercise. In addition, dynamic muscle performance involving the stretch-shortening cycle changed more in parallel with soreness than static measures during the recovery from eccentric exercise.	High-intensity eccentric exercise has been shown to result in muscle damage, with symptoms including DOMS, strength loss, restricted ROM and increased blood protein.
	Sarac et al. (36)	36 participants Age 19–21 years old	Pain and fatigue levels using a visual analog scale and assessed skin surface temperature over the quadriceps femoris muscle with thermal imaging	Parallel, single-blinded, randomized controlled trial; 3 Groups: - Percussive massage group (*n* = 12), - Foam roller group (*n* = 12), Control group (*n* = 12) Fatigue protocol for quadriceps femoris was performed. Participants received soft tissue mobilization with foam roller/percussive massage or rested for 10 min according to their groups Pain and fatigue were evaluated by a visual analog scale, Skin surface temperature of quadriceps femoris was measured with thermal camera imaging Evaluations were performed pre fatigue protocol, post at 24th hour and at 48th hour Changes from the baseline at 24th and 48th hours were compared between groups Protocol fatigue = duration?	No significant differences were found between the groups before and after the fatigue protocol (at 24 and 48 h). Soft tissue mobilizations using a foam roller or percussive massage device do not appear to be more effective than passive rest in reducing DOMS symptoms in recreational athletes.	The lack of efficacy might be due to the neurophysiological mechanisms underlying DOMS, which may not be sufficiently influenced by these soft tissue mobilization techniques. They proposed that DOMS involves complex neurophysiological processes, including muscle microtrauma and inflammation, which might not be effectively mitigated by foam rolling or percussive massage
	Sieljacks et al. (52)	14 young untrained males. Age 23–27 years old	- Muscle Cross-Sectional Area (CSA) with Magnetic resonance imaging (MRI) of the quadriceps and its components (vastus lateralis, vastus medialis, vastus intermedius, and rectus femoris) before and after the training period. - MVIC - Isokinetic strength and strength-endurance capacity (3-RM) were evaluated pre- and post-training. - RPE - DOMS	- 8-week training program where each leg was randomly assigned to one of two protocols: - Failure blood flow restricted exercise (F-BFRE): One leg did unilateral knee extensions to failure at 25% of 1-RM with 40% AOP cuff pressure. - Non-Failure BFRE (NF-BFRE): The other leg completed knee extensions at the same load for 75% of the reps from the F-BFRE session. - 22 sessions assessed muscle CSA, strength, and perceptions before and after training.	Both F-BFRE and NF-BFRE significantly increased muscle CSA and strength, with no major differences between them. NF-BFRE had lower ratings of perceived exertion, discomfort, and DOMS than F-BFRE, with DOMS reported only after the first F-BFRE session; no DOMS was noted in either leg after the second last session.	Fatigue significantly influences perceptual responses during BFRE. Higher perceived exertion and discomfort during F-BFRE are linked to increased metabolic stress and muscle fatigue. This enhanced fatigue likely activates pain-related group III and IV afferent fibers. The lack of DOMS in NF-BFRE may result from lower fatigue and muscle damage, as fatigue drives perceived discomfort. The study suggests that non-failure BFRE can still effectively stimulate muscle adaptations while reducing discomfort, making it suitable for clinical populations.
	Shimomura et al. (53)	14 males, and 16 females. Age 21–24 years old	Fatigue VAS	Branched chain amino acids (BCAA) Green Tea powder	BCAA decreased DOMS and muscle fatigue for a few days after exercise, attenuated muscle protein breakdown, and postexercise muscle protein synthesis was greater	Muscle protein breakdown.
	Stock et al. (54)	17 males and 3 females. Average age 22 years old	CK LDH	Leucine added to a carbohydrate beverage	These results do not support adding leucine to carbohydrate beverages before and after resistance training within the context of this study.	Damage to myofibrillar and cytoskeleton proteins, increase in serum CK and LDH.
	Tenberg et al. (27)	7 males and 4 females healthy participants	- Maximal Voluntary - Isometric Contraction (MVIC) - Pressure Pain Threshold (PPT) - Palpation Pain - Extramuscular Connective Tissue (ECT) Thickness - ECT and Muscle Displacement	A randomized controlled crossover trial Two exercises types : Eccentric Exercise (EE) and Concentric Exercise (CE) of 80% of the individual 1-RM. Each participant underwent both exercise conditions with a minimum of 72 h between sessions Measures were taken before, immediately after, and at intervals up to 96 h post-exercise.	- Palpation Pain: Greater after EE compared to CE, indicating more soreness associated with eccentric exercise. - MVIC Torque: Decreased significantly more after EE than CE, suggesting greater muscle fatigue and damage. - ECT Thickness: Increased significantly after EE at 48, 72, and 96 h post-exercise, with a correlation between increased ECT thickness and palpation pain at 96 h. - Muscle Displacement: Increased after EE, indicating altered mobility of the muscle tissue. The changes in ECT morphology may contribute to the pathogenesis of DOMS, although a direct cause-effect relationship was not established	The ECT has a high nociceptive capacity due to its dense innervation and presence of sensory receptors. The increased ECT thickness following eccentric exercise is hypothesized to reflect tissue inflammation and/or edema, possibly triggered by micro-damage to collagen fibers in the connective tissue. This micro-damage can lead to increased pain sensitivity, as evidenced by the correlation between ECT thickness and palpation pain. Pain responses are often stronger when the connective tissue is irritated compared to muscle tissue, suggesting that the fascia (ECT) plays a significant role in the sensation of pain associated with DOMS
	Weber et al. (15)	40 females. Age 18–35 years old	Maximal Voluntary Isometric Contraction (MVIC)	Therapeutic massage upper body ergometry microcurrent electrical stimulation	The results of the present study did not provide support for the use of massage, microcurrent electrical stimulation, or upper body ergometry (concentric exercise) applied immediately following and 24 h after a high-intensity eccentric exercise bout to reduce or alleviate soreness and force deficits associated with delayed onset muscle soreness.	One theory suggests that DOMS and the associated force deficit are caused by damage to the muscle fibres and the intramuscular connective tissue with myofibril damage, particularly of the Z-band or connective tissue damage at the distal musculotendinous junction.
	Wernbom et al. (55)	8 males and 3 females. Age 20–39 years old	Quadriceps muscle activity using electromyography (EMG) and assessed endurance by recording the number of repetitions performed until concentric torque failure at 30% of (1RM).	Blood flow restriction (BFR)	While BFR did not significantly increase maximum muscle activity compared to non-occluded exercise, it did decrease endurance, as evidenced by fewer repetitions performed under BFR conditions	Reduced endurance with BFR might be due to accelerated muscle fatigue resulting from restricted blood flow, leading to an earlier onset of muscle exhaustion
Plyometrics	Adamczyk et al. (33)	36 physically active male participants. Age 20–27 years old	- Thermal Imaging (lower limbs; at rest, immediately after exercise, after recovery treatment (ice massage or cold-water immersion), and after 30 min of rest). - Blood Lactate Levels (at rest and again 30 min post-exercise). - DOMS (pain levels using VAS at 24, 48, 72, and 96 h post-exercise)	- 3 groups (*n* = 12/group): Ice Massage (IM) (a 3-min ice massage on the quadriceps muscles immediately after exercise), Cold-Water Immersion (CWI) (lower limbs immersed in water at 8 °C for 3 min immediately after exercise), and Passive Recovery (PAS) (control group rested passively). - After a one-minute squat jump exercise, recovery treatments were applied. IM and CWI effectiveness was assessed through lactate clearance, temperature changes, and DOMS-related pain levels.	- Lactate Clearance: IM and CWI lowered blood lactate significantly compared to PAS. - Temperature Changes: Both treatments decreased skin temperature, with CWI showing a greater drop. After 30 min, skin temperatures in IM and CWI were near resting values, while PAS returned to resting. - Pain Reduction: VAS scores showed significant pain relief in IM and CWI groups vs. PAS, especially at 72 and 96 h post-exercise.	- Cold therapy initially causes vasoconstriction, reducing blood flow and metabolism and decreases inflammation from muscle microdamage, a key cause of DOMS. Cold exposure lowers nerve excitability and pain perception by increasing β-endorphin release and reducing nociceptive signals. The combined effects of lower temperature and enhanced blood flow speed up muscle recovery and reduce soreness.
	Cardoso et al. (56)	45 males and females. Age 18–40 years old	- DOMS using a VAS -PPT (by pressure algometer)	- 3 key phases: Pre-intervention phase, Exercise-Induced Muscle Damage (EIMD) Protocol (drop jumps from a box repeated over several sets to create significant muscle strain) and Intervention phase with 3 groups: - Verum Acupuncture Group (VA): acupuncture at specific points (ST34, ST36, LR3) on the non-dominant lower limb. - Sham Acupuncture Group (SA): acupuncture at 3 non-traditional points on the non-dominant lower limb. - Control Group (CG): without treatment, simply rested. - The acupuncture treatment was administered for 2 min immediately after the initial assessments (T1 and T3) following the exercise-induced muscle damage protocol	- VA and SA significantly reduced Acute Muscle Soreness (AMS). VA has improved more than SA and CG. - VA reduced DOMS by one-third while no effects were observed in the SA and CG. - The DOMS prevention was not significantly affected by previous treatments in either the VA or SA groups.	- Acupuncture may create microinjuries that enhance local blood flow, facilitating healing and producing analgesia through the stimulation of nerves, leading to the release of neuropeptides like encephalin and endorphins. It can reduce pain in the segment where needles are inserted, as the action potentials travel up the nerve to the spinal cord, decreasing the dorsal horn's response to painful stimuli. - Even though sham acupuncture was applied at non-traditional points, it may still have activated reflex effects or acted as trigger points, contributing to pain relief. - The release of substances like bradykinin and calcitonin gene-related peptide activate muscle nociceptors and may contribute to the perception of soreness.
	Chatzinikolaou et al. (4)	24 males. Average age 27 years old	PPT Knee range of motion, CK lactate dehydrogenase white blood cell count, CR) uric acid cortisol testosterone IL-6, IL-1b strength and jumping performance	None	An acute, intense plyometric exercise may induce short-term muscle damage and marked but transient inflammatory responses. Jumping performance seems to deteriorate after 72 h post exercise, whereas strength remains unchanged.	Reductions of force output and ROM by the injured muscle and edema that persist for several days. Muscle damage is mainly induced by mechanical stress and disturbances of calcium homeostasis.
	Huang et al. (57)	36 untrained males. Age 21.09 ± 1.33 years old	- Exercise Performance Tests (CMJ, Wingate Anaerobic Test (WAnT)) - Muscle Soreness Assessment : Pressure Pain Threshold (PPT) - Biochemical Markers: creatine kinase (CK), lactate dehydrogenase (LDH), liver and renal function indicators. - Complete Blood Count (CBC)	A double blind, placebo-controlled trial.	Resveratrol Supplementation (RES) supplementation significantly reduced muscle damage and soreness following PEIMD, effectively mitigated the increase in muscle soreness and accelerated recovery from DOMS.	RES reduce oxidative stress by neutralizing free radicals and boosting antioxidant enzymes, protecting muscle fibers during intense exercise. It downregulates inflammatory pathways like NF-κB, reducing inflammation, pain, and muscle nociceptor sensitivity. RES promotes muscle recovery by enhancing satellite cell regeneration and minimizing enzyme leakage (e.g., CK, LDH) into the bloodstream. Additionally, RES supports muscle performance and strength post-exercise by improving neuromuscular function and reducing fatigue during recovery.
				3 groups (*n* = 12/group): placebo (1,000 mg/day of methylcellulose), Resveratrol Supplementation RES-500 (500 mg RES/day + 500 mg/day methylcellulose), and RES-1000 (1,000 mg RES/day)		
				Supplemented for 10 days prior to undergoing the plyometric exercise-induced muscle damage (PEIMD) protocol, which involved performing 10 × 10 maximal vertical jumps.		
	Váczi et al. (58)	8 males. Average 21 years old	Maximal isometric voluntary torque (MVIC) and Rate of torque development (RTD) Unilateral vertical jump height (VJ) CK Acute lactate Heart rate responses	None	The results suggest that, although vigorous stair-jump training acutely produces fatigue in trained individuals, it does not compromise power and rapid torque-generating ability on a subsequent day and could be one alternative to prevent muscle soreness. Due to its non-damaging nature, stair jumps could be incorporated into the beginning of the pre-season preparation when practitioners return from the off-season. However, It is suggested that coaches and strength specialists use such unilateral exercises (especially at level) with special caution, as they may increase the mechanical stress on the spine or the lower extremity joints.	Temporary muscle damage.increased oxygen consumption, heart rate, and blood lactate.
	Wahl et al. (14)	20 males. Age 24 years old	Extensor muscles Creatine kinase (CK) Lactate dehydrogenase (LDH) Myoglobin PEPS	Aqua cycling	The results of this study showed that a single 30-minute Aqua cycling session after 300 countermovement jumps did not affect the recovery of muscular performance, the increase in markers of muscle damage, muscle soreness, or the PEPS compared with passive rest.	Induction of edema and metabolites. CK, LDH and myoglobulin.

Quantitative summary: in summary, effect sizes are reported by only 9 studies (17, 28, 38–44), and they show trends toward DOMS reduction with positive effects evident for acupuncture (56, 59) and compression (23, 46, 60, 72). However, scarcity of quantitative data from all but 9 primary studies, and non-standardized metrics, critically weaken robust pooled estimates and temporal synthesis.

### 2.2 Eligibility criteria

#### 2.2.1 Inclusion criteria

Studies were included if they:

- Were published in English in peer-reviewed journals
- Focused on neurophysiological aspects of DOMS, including imaging modalities and biomarkers
- Were RCTs, controlled trials, or prospective cohort studies
- Included participants engaged in endurance, resistance, or plyometric exercise
- Studies involving both trained and untrained participants were considered eligible, as training status may influence the magnitude of DOMS, neuromuscular adaptations, and recovery responses.

#### 2.2.2 Exclusion criteria

Studies were excluded if they:

- Were systematic reviews, meta-analyses, case reports, or opinion papers (*n* = 15)
- Did not present original primary data
- Focused on non-specific muscle soreness without relevance to DOMS
- Lacked neurophysiological outcome measures

### 2.3 Study selection process

The PRISMA flow diagram is presented in Figure 2.

The selection process was conducted in six phases:

1. Identification: 1,183 records were retrieved.
2. Screening: Duplicates and clearly irrelevant studies were removed, leaving 131 articles.
3. Title and Abstract Review: Two independent reviewers screened all records against eligibility criteria.
4. Inter-Rater Reliability: Agreement between reviewers during screening was calculated using Cohen's kappa coefficient (*κ*), demonstrating substantial agreement (*κ* = 0.82). Discrepancies were resolved through discussion and, when necessary, consultation with a senior researcher.
5. Full-Text Review: 68 studies met full-text eligibility criteria.
6. Data Extraction: Data extraction was conducted independently by the reviewers using a standardized extraction template capturing study design, population, intervention characteristics, outcome measures, and key findings. All reviewers had access to the open access excel spreadsheet template.

### 2.4 Quality assessment

Methodological quality was assessed independently by two reviewers using the Newcastle-Ottawa Scale (NOS). Disagreements were resolved by consensus. Risk of bias results are presented in Table 3.

## 3 Results

An overview of reported outcomes across endurance, resistance, and plyometric exercise modalities is provided in Table 1.

### 3.1 Neurophysiology of DOMS

The included studies investigated neurophysiological processes associated with DOMS using biochemical, electrophysiological, and functional outcome measures (1, 3, 8, 36, 46–63).

Reported outcomes included changes in electromyographic activity, temporary reductions in voluntary force production and range of motion, and increased pain sensitivity during the post-exercise period (8, 25, 63). Biochemical indicators related to inflammatory and metabolic processes were also assessed, although the magnitude and temporal characteristics of these responses varied substantially across studies and exercise protocols (1, 3, 28, 36, 46).

#### 3.1.1 Damage to myofibrils and connective tissue

Our findings suggest that DOMS results from mechanical disruption of myofibrils and connective tissue (10, 12, 15) caused by the high tension associated with eccentric contractions. During eccentric exercise, high forces are concentrated on a subset of fast-twitch muscle fibers, leading to disruption of noncontractile structures. This stress can induce sarcomere disorganization, Z-line to M-line perturbation, and rupture of the sarcolemma and sarcoplasmic reticulum (31). The resultant disturbance of calcium homeostasis (41, 61) and activation of phospholipase-prostaglandin and calpain pathways can exacerbate muscle damage and nociceptor activation (9, 18).

Electron microscopy confirms structural myofibril damage in DOMS-induced muscle samples, particularly at the Z-line (46). Furthermore, impaired antioxidant capacity contributes to oxidative stress-induced muscle damage (6, 7), leading to increased reactive oxygen species (ROS) production and exacerbating muscle fiber disruption.

#### 3.1.2 Inflammatory response

Muscle damage triggers a secondary inflammatory cascade, marked by muscle fiber swelling, mast cell degranulation, degradation of intracellular structural proteins, and neutrophil infiltration (12, 46). Acute transient inflammation is beneficial for muscle repair (28), but excessive or prolonged inflammation can delay recovery (31). The resolution of inflammation is essential for transitioning from muscle damage to tissue repair. The finding showed a variety of inflammatory response's biomarkers, as the following:

##### 3.1.2.1 Cytokines

DOMS is linked to an acute inflammatory process characterized by elevated cytokine levels (IL-6, IL-1β, TNF-α) (4, 28, 29, 44) following eccentric contractions. Key cytokines such as IL-1β and IL-6 regulate immune responses, with IL-6 playing a dual role in promoting both inflammation and repair (4). Anti-inflammatory IL-10 modulates inflammation by inhibiting IL-6 and IL-1β production (31).

##### 3.1.2.2 Leukocytes

Leukocyte infiltration is a hallmark of exercise-induced inflammation, peaking 2–3 days post-exercise. Neutrophils and monocytes infiltrate damaged muscle, releasing free radicals and inflammatory mediators, which may prolong DOMS-associated soreness (4, 31).

##### 3.1.2.3 Serum proteins

Elevated levels of creatine kinase (CK), lactate dehydrogenase (LDH), and myoglobin serve as indirect markers of muscle tissue damage (14, 38). CK levels typically peak 24–72 h post-exercise and contribute to strength deficits (6), whereas LDH correlates with exercise intensity (4, 36).

##### 3.1.2.4 Cortisol

Exercise modulates cortisol levels, which serve as an indicator of physiological stress. Cortisol elevation depends on exercise intensity, duration, and glycogen availability, with high levels potentially exacerbating DOMS by influencing muscle protein degradation and inflammation (4, 46).

##### 3.1.2.5 Oxidative stress

Exercise-induced muscle damage is associated with increased ROS production (6, 7) and reduced antioxidant defenses, leading to lipid peroxidation and mitochondrial dysfunction (31, 41). While oxidative stress plays a role in muscle recovery, excessive ROS may contribute to prolonged soreness and impaired muscle regeneration (4).

##### 3.1.2.6 Calcium channels

Intracellular calcium dysregulation (10, 41, 61) contributes to DOMS-associated muscle fatigue and soreness. Overstretched sarcomeres allow excessive calcium influx, which activates proteases that degrade muscle proteins (4, 41). This disrupts sarcoplasmic reticulum function, leading to prolonged contracture and nociceptor activation (56).

##### 3.1.2.7 Edema

Exercise-induced edema results from increased vascular permeability and fluid accumulation in the interstitial space, causing increased intramuscular pressure and nociceptor activation (41, 48). Prostaglandins released via the cyclooxygenase pathway increase vascular permeability, vasodilation and sensitize pain receptors (56).

##### 3.1.2.8 Pain catastrophizing

Psychological factors, such as pain-related fear and kinesiophobia, may influence DOMS severity (9). Temporal summation of pain (TSP) and conditioned pain modulation (CPM) mechanisms may predispose individuals to heightened pain sensitivity, affecting recovery rates.

##### 3.1.2.9 Central sensitization mechanisms

DOMS induces spinal cord hyperexcitability through temporal summation of pain and c-Fos upregulation in dorsal horn neurons (18, 19). Group III/IV afferents from sensitized nociceptors trigger glutamate/NMDA receptor activation with reduced descending inhibition (19). fMRI evidence reveals anterior cingulate cortex and insula hyperactivation during DOMS (18).

##### 3.1.2.10 Psychological predictors of DOMS severity

Psychological factors appear to meaningfully influence the severity and functional impact of DOMS. Pain catastrophizing has been shown to predict greater DOMS intensity, while kinesiophobia correlates with increased maximal voluntary contraction (MVC) loss following exercise (9). Because DOMS mimics mild musculoskeletal pain, it may activate fear-related responses consistent with the Fear-Avoidance Model, whereby fear of pain leads to movement avoidance, reinforcing perceived pain, disability, and reductions in physical activity. Hypervigilance and catastrophizing have been associated with higher post-exercise pain intensity and increased likelihood of DOMS development (9). These psychological processes may interact with physiological mechanisms, potentially contributing to heightened central sensitisation and altered pain modulation. Clinically, identifying individuals with elevated fear, catastrophizing, or hypervigilance may allow for early, personalised interventions aimed at reducing maladaptive pain responses and preventing unnecessary movement avoidance or pain chronification.

##### 3.1.2.11 Clinical translation

Cognitive-behavioral interventions demonstrate efficacy in reducing catastrophizing and restoring function (9). High catastrophizers exhibit amplified peripheral sensitization and poorer response to peripheral therapies including compression (9, 60).

From a clinical perspective, these findings suggest that DOMS management should extend beyond treating pain alone. Clinicians, physiotherapists, sports physicians, strength and conditioning coaches, and rehabilitation practitioners should consider the underlying neurophysiological mechanisms together with exercise modality, training load, recovery timing, and individual responsiveness when selecting recovery strategies. Such an individualized approach is likely to optimise recovery while minimising unnecessary interruption of training or rehabilitation.

### 3.2 Measures of DOMS

The assessment of DOMS incorporates a combination of subjective and objective measures to evaluate pain, inflammation, muscle function, and biochemical markers of muscle damage. Because pain perception is influenced by both peripheral nociceptive input and central processing mechanisms, subjective pain ratings should ideally be interpreted alongside objective measures of muscle function, range of motion, swelling, and neuromuscular performance. Recent evidence from chronic pain research further highlights that pain intensity does not always correlate with the extent of tissue pathology, emphasizing the importance of multidimensional assessment approaches (9, 35, 105).

#### 3.2.1 Visual analog scale (VAS)

Self-reported questionnaires are commonly employed to subjectively quantify the intensity of soreness associated with DOMS (43). Among these, the VAS is one of the most widely used tools for pain assessment. It provides a continuous scale on which individuals rate their perceived pain intensity. However, VAS is inherently subjective and can be influenced by individual pain perception, cognitive biases, and psychological factors (34). Consequently, it is recommended that VAS be supplemented with objective physiological parameters to enhance the reliability of DOMS assessment (56).

#### 3.2.2 Pressure pain threshold (PPT)

Pressure-based pain assessments help determine localized mechanical sensitization in response to DOMS (9). PPT measures the minimum force required to induce discomfort, reflecting muscle sensitivity to pressure and nociceptor activation (34). While PPT is considered a more objective measure than VAS, its variability among individuals may limit consistency in findings (56). Notably, baseline pressure tolerance is inversely related to DOMS severity, with individuals exhibiting higher-pressure tolerance experiencing less post-exercise muscle soreness (9).

#### 3.2.3 Limb circumference and muscle swelling

Limb circumference measurements serve as an indirect assessment of muscle swelling and edema resulting from sarcomere disruption and inflammatory responses (43). Swelling is attributed to the accumulation of protein-bound ions, which exert osmotic pressure within muscle tissue, leading to extracellular fluid retention. However, some studies have reported no significant increase in limb circumference following eccentric exercise, highlighting variability in edema responses (6). Imaging techniques such as ultrasound and MRI provide higher accuracy in quantifying muscle edema and structural changes than circumferential measurements (41).

#### 3.2.4 Microscopy and muscle fiber disruption

Histological analysis through light and electron microscopy offers direct visualization of muscle damage following eccentric contractions. DOMS is associated with inflammatory cell infiltration, disruption of Z-disks, and sarcolemma rupture, which have been correlated with the severity of perceived muscle soreness (41). While microscopy provides a precise assessment of muscle damage, it is invasive and primarily used in research settings rather than routine clinical evaluation.

#### 3.2.5 Biochemical markers of muscle damage

Biochemical assays are used to evaluate muscle damage, inflammation, and metabolic stress associated with DOMS. Among the most commonly measured markers are:

- Creatine Kinase (CK)—A widely recognized marker of muscle fiber disruption and membrane permeability changes (13). CK levels typically peak 24–72 h post-exercise and remain elevated during recovery, though CK does not always correlate with perceived soreness (45).
- Lactate Dehydrogenase (LDH)—Released into the bloodstream following muscle cell damage, LDH levels increase post-exercise and are useful in identifying the extent of muscle strain (14).
- Myoglobin (Mb)—A muscle-specific protein that rises rapidly in circulation following acute muscle injury, serving as an early indicator of exercise-induced muscle damage (14).
- Interleukin-6 (IL-6) and C-Reactive Protein (CRP)—Markers of systemic inflammation, with IL-6 acting as both a pro-inflammatory and anti-inflammatory cytokine in response to muscle stress (17).

#### 3.2.6 Muscle function: joint torque and range of motion (ROM)

DOMS is associated with temporary impairments in muscle force output and joint mobility due to pain, muscle stiffness, and inflammation (4). Measures of joint torque and ROM provide objective insights into the neuromuscular impact of DOMS:

- Maximal Voluntary Contraction (MVC)—Studies indicate a 20%–60% decline in MVC post-eccentric exercise, serving as a valid and reliable marker of muscle strength loss (41).
- Isometric and Isokinetic Torque Testing—Provides precise quantification of muscle force generation capacity and fatigue resistance in DOMS-affected muscles (10).
- Goniometry—Used to assess reductions in ROM, which may result from muscle stiffness and spontaneous shortening post-exercise (11, 43).

Clearly, the assessment of DOMS requires a multifaceted approach combining subjective pain perception (VAS, PPT), physiological markers (muscle swelling, biochemical assays), functional performance (MVC, torque measurements), and structural evaluations (microscopy, imaging). Given the variability in individual responses, integrating multiple assessment tools enhances accuracy and reliability in both research and clinical settings.

### 3.3 Neurophysiological variations in DOMS across exercise modalities

#### 3.3.1 Endurance-related DOMS

Several studies have reported DOMS following endurance-based exercises, highlighting its physiological and biochemical implications as can be seen in Table 1 (29–36). However, only one study has specifically investigated the neurophysiological mechanisms underlying endurance-related DOMS. Jafariyan et al. (31) examined inflammatory and immune responses over a three-day period of repeated bouts of downhill running in active females. The experimental protocol involved three separate 60 min downhill running sessions, each separated by 24 h, with data collected 2 h post-exercise for each bout.

The authors reported an increase in DOMS intensity across multiple muscle groups. Notably, quadriceps soreness intensified after the second and third bouts, while gluteus and gastrocnemius soreness increased after the second bout, resolving 2 h after the final session. This delayed pain response aligns with the repeated bout effect, wherein prior exposure to eccentric or unaccustomed exercise results in a protective adaptation that mitigates subsequent muscle damage and soreness (31).

In terms of biochemical markers, significant increases were observed in serum CK, myoglobin (Mb), and LDH following each exercise bout, with CK and Mb levels being significantly higher after the third bout compared to the first. These findings suggest an accumulative muscle stress response to repeated endurance exercise. Additionally, blood white cell count increased after the first two bouts, while neutrophils and monocytes consistently rose after all three bouts, and lymphocyte levels declined after the second session.

From an immunological perspective, key inflammatory cytokines were altered. Serum IL-10 levels increased after each exercise bout, peaking after the first session. Monocyte chemoattractant protein-1 (MCP-1) and interleukin-6 (IL-6), both of which are involved in the acute inflammatory response to muscle damage, showed significant elevations post-first bout, further supporting the notion of an initial inflammatory surge that decreases with subsequent exposures. Furthermore, knee extensor strength was consistently reduced across all exercise bouts, but the decline was only statistically significant after the first two sessions. Strength levels slightly improved before the third bout, likely due to the body's adaptive protective mechanisms against exercise-induced damage (31).

While, Table 1 summarizes the distinct effects of endurance, resistance, and plyometric exercise on DOMS and their respective neurophysiological mechanisms, Table 2 provides further insights into the specific neurophysiological pathways associated with each exercise type. Although endurance exercise primarily induces metabolic stress rather than mechanical overload, it still elicits muscle damage, inflammatory responses, and neuromuscular impairments, similar to resistance and plyometric training. In summary, the primary mechanisms implicated in endurance-related DOMS include:

- Inflammation-mediated sensitization of nociceptive afferents, particularly due to increased IL-6, MCP-1, and IL-10 (31).
- Membrane permeability changes and increased oxidative stress, leading to calcium dysregulation and activation of proteolytic pathways, which contribute to muscle fatigue and soreness (30).
- Delayed force recovery, attributed to neuromuscular fatigue and central inhibition following prolonged eccentric muscle actions (32).
- The repeated bout effect, providing a degree of protection against further damage via enhanced neuromuscular coordination and muscle remodeling (38).

**Table 2:** Neurophysiological mechanisms of DOMS related to exercise type.

Mechanism	Resistance-related DOMS	Plyometric-related DOMS	Endurance
Exercise type	High-intensity or novel resistance training (eccentric contractions)	High-intensity, explosive movements (stretch-shortening cycle)	Prolonged aerobic activities involving repetitive, submaximal muscle contractions.
Primary mechanism	Muscle microtrauma, particularly in eccentric contractions	Rapid muscle lengthening and stretch-shortening cycle, leading to microtrauma	Repetitive loading induces microscopic tears in muscle fibers
Nociceptor activation	Group III and IV afferents (mechanical and chemical stimuli)	C-fibers and Aδ afferents (mechanical and chemical stimuli)	Accumulation of acidic metabolites lowers pH i.e., hydrogen ions (H⁺). potassium (K⁺) released from damaged muscle cells
Inflammatory mediators	Cytokines (IL-6, TNF-α), prostaglandins, bradykinin	Prostaglandins, bradykinin, interleukins, oxidative stress	Cyclooxygenase (COX) pathway activation leads to prostaglandin synthesis. Neutrophils and macrophages amplify inflammation.
Peripheral sensitization	Increased nociceptor sensitivity (due to prostaglandins, bradykinin)	Enhanced nociceptor activation via inflammatory mediators and oxidative stress	Prolonged sensitization maintains localized pain and tenderness.
Central sensitization	Spinal cord hyperexcitability (allodynia), increased excitatory neurotransmitters (glutamate)	Upregulation of c-Fos in dorsal horn, central sensitization leading to hyperalgesia	Increased release of glutamate and substance P in the spinal cord.
Proprioception and movement control	Altered proprioception, impaired motor control, reduced range of motion and strength	Impaired motor unit activation, reduced neuromuscular efficiency, increased antagonist coactivation	Impaired proprioceptive feedback from muscle spindles and Golgi tendon organs.
Pain perception modulation	Reduced efficiency of descending pain modulation (serotonin, norepinephrine)	Impaired descending inhibitory pathways, altered pain modulation (serotonin, endorphins)	Endogenous opioid release descending inhibition involving the PAG-RVM axis
Recovery and adaptation	Inflammatory response aids muscle repair, improves resilience	Proprioceptive feedback, motor unit synchronization, and stretch-shortening cycle efficiency improve over time	Increased mitochondrial biogenesis and oxidative enzyme activity.
CNS adaptation	Enhanced neuromuscular function with repeated training, decreased pain sensitivity	Improved motor unit synchronization, reduced antagonist coactivation, better proprioception with repeated training	Enhanced connectivity in pain modulation networks, such as the periaqueductal gray (PAG) and rostral ventromedial medulla (RVM), results in better descending pain inhibition.

To conclude, although endurance exercise shares several neurophysiological similarities with resistance and plyometric exercises, no conclusive evidence has been found to suggest that the mechanisms of DOMS following endurance exercise differ fundamentally from other exercise modalities.

#### 3.3.2 Neurophysiology of the therapeutical interventions that ease endurance-related DOMS

A total of 35 studies were identified that examined various interventions aimed at alleviating DOMS, with 22 of these studies demonstrating beneficial effects. Table 3 and Figure 3 provide summaries of the therapeutic approaches used and their proposed neurophysiological mechanisms underpinning treatment effectiveness.

**Table 3:** Neurophysiological mechanisms contributing to DOMS relief.

Intervention	Neurophysiological mechanisms	Effect on DOMS
Neurodynamic mobilization (NM)	Reduces neural tension	Alleviates neural irritation
	Improves blood circulation	Reduces muscle soreness and stiffness
Foam rolling	Enhances circulation	Decreases pain perception
	Decreases muscle inflammation	Reduces muscle stiffness
	Reduces muscle adhesions	Eases soreness and accelerates recovery
	Enhances lymphatic drainage	
Blood flow restriction (BFR)	Enhances metabolic and circulatory responses	Reduces severity of soreness
	Promotes muscle growth and recovery despite reduced mechanical stress	Speeds up recovery by improving muscle adaptation
Manual therapies (e.g., massage)	Enhances local circulation	Alleviates muscle soreness
	Reduces muscle inflammation	Reduces stiffness and improves recovery times
Low-level laser therapy	Increases blood flow	Helps decrease pain and inflammation
	Stimulates cellular repair mechanisms	Accelerates recovery
Electrical stimulation (NMES)	Enhances circulation	Decreases neural hypersensitivity
	Reduces central nervous system (CNS) fatigue	Speeds up recovery and reduces pain
	Restores normal motor neuron excitability	Improves neuromuscular recovery
	Enhances motor unit recruitment	
	Reduces muscle fatigue	
Cryotherapy	Reduces inflammation	Reduces pain perception
	Attenuates afferent sensitization (Group III and IV fibers)	Reduces stiffness, acute pain and swelling
	Lowers nerve conduction velocity	
Compression garments	Decreases local inflammation	Reduces soreness and muscle stiffness
	Reduces group III and IV afferent sensitization	Enhances recovery by reducing inflammation
	Modulates muscle stiffness, limits edema	No significant effect on muscle perfusion
Pharmacological approaches	Modulates peripheral and central nociceptive	Variable efficacy
	pathways (e.g., NSAIDs, serotonin reuptake inhibitors) which block prostaglandin-mediated pain pathways	Reduces peripheral and central sensitization
		Decreases pain and sensitivity to mechanical stress
PBMT photobiomodulation therapy (PBMT)	Increases mitochondrial ATP production	Enhances muscle regeneration

**Figure 3:**
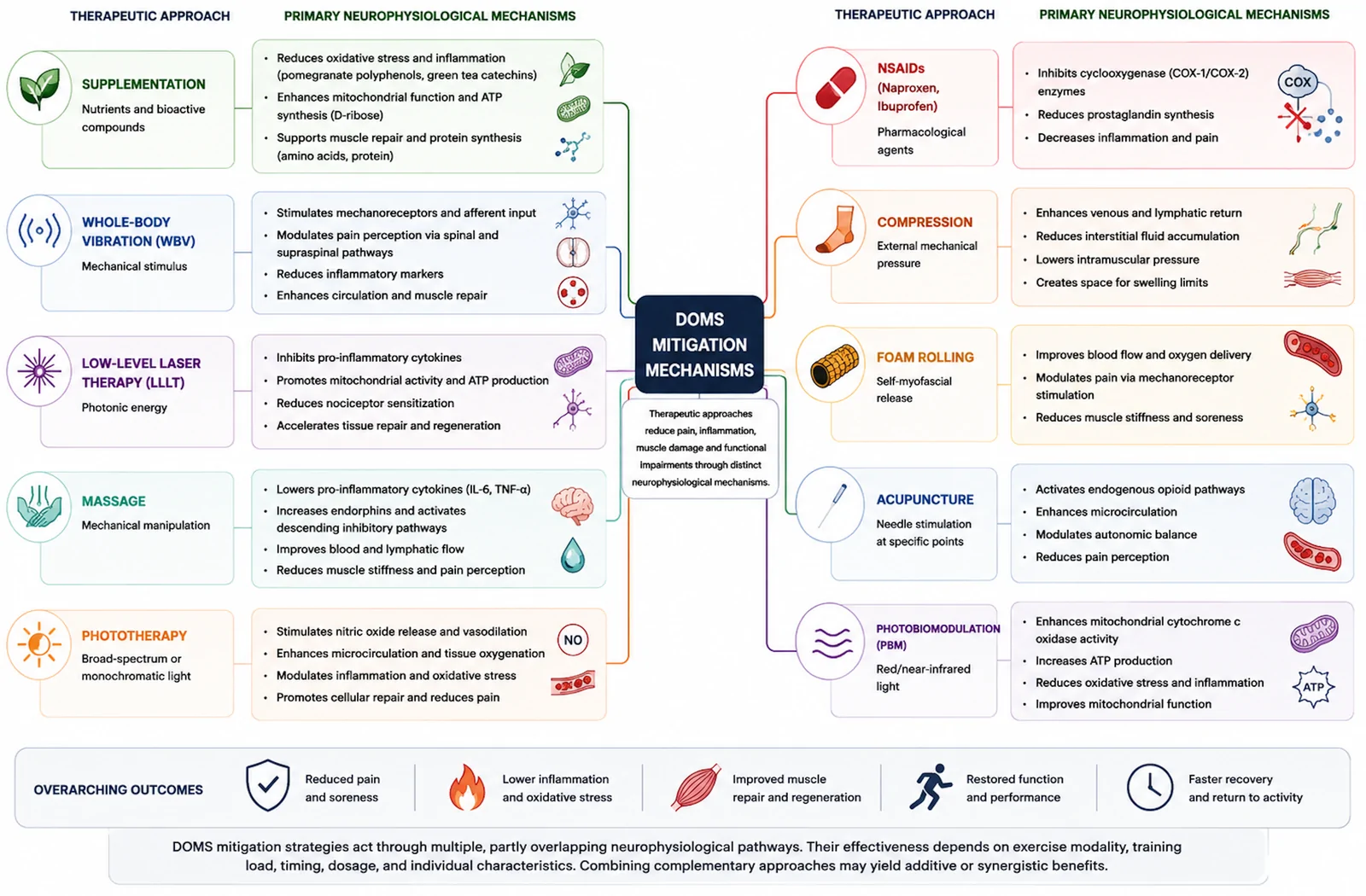
Summaries of the therapeutic approaches and their proposed neurophysiological mechanisms underpinning treatment effectiveness for DOMS.

##### 3.3.2.1 Neuromuscular stimulation and mechanical interventions

- Whole Body Vibration (WBV) increased motor-unit activity, slow-fiber recruitment, and motor-unit synchronization by enhancing gamma motor neuron activation, muscle spindle sensitivity, and neuromuscular facilitation (6).
- Massage therapy alleviated DOMS by reducing CK and IL-6 levels, increasing blood and lymphatic flow, and enhancing skin temperature, which accelerates CK clearance and removes immune cells from the injured site, reducing further muscle damage (17, 64).
- Foam rolling reduced DOMS through:
  1. Decreasing muscle swelling,
  2. Enhancing lactate clearance via increased blood flow,
  3. Mitigating inflammation by preventing leukocyte adhesion to endothelial walls

Additionally, foam rolling has been shown to lower prostaglandin levels and increase oxygen supply, which aids in muscle repair and recovery. Similar to massage, it can also increase white blood cell count, activate mitochondrial biogenesis, and reduce cell stress proteins (8).

##### 3.3.2.2 Nutritional and supplementation-based interventions

- Pomegranate supplementation, rich in polyphenols, alleviated DOMS by reducing tissue edema and metabolic by-products, with effectiveness dependent on dosage and muscle mass volume recruited (65).
- D-ribose supplementation promoted faster muscle cell membrane repair, reducing CK and Mb leakage into the bloodstream. Additionally, D-ribose played a role in salvaging cellular energy, reducing oxidative stress, and decreasing malondialdehyde (MDA), a marker of oxidative damage (66).
- Green tea supplementation, high in catechins, improved energy utilization through increased fat oxidation and higher GLUT-4 protein expression, which facilitates glucose uptake into muscle cells. The reduction in CK levels suggests a shift in metabolic substrate utilization, lowering reliance on anaerobic energy sources (7).
- Amino acid supplementation significantly reduced muscle damage markers when consumed post-exercise. Essential amino acids (EAAs) contributed to protein synthesis enhancement despite no significant changes in CK or protein carbonyl levels (38, 45).
- NPN_1 supplementation reduced DOMS by:
  - Lowering myostatin (a negative regulator of muscle growth),
  - Decreasing FGF21 (linked to muscle stress),
  - Increasing irisin (involved in energy storage in muscles) and IL-15 (associated with muscle growth and repair),
  - Enhancing phosphorylation of S6 kinase, a crucial regulator of protein synthesis and muscle hypertrophy (28).
- Resveratrol supplementation (RES), a potent antioxidant, alleviated DOMS by reducing CK, LDH, oxidative stress markers, and lipid peroxidation-related damage (57).

##### 3.3.2.3 Pharmacological interventions

- NSAIDs provided inconsistent relief from DOMS. Some studies demonstrated benefits by reducing nociceptive signaling through inhibition of prostaglandins and cyclooxygenase (COX) enzymes (67, 68). However, other studies found no significant effects on DOMS (69, 70).
- Naproxen, an NSAID, was associated with a reduction in RI-T2 signal intensity on MRI, an indicator of muscle damage and inflammation, particularly in the quadriceps (41).

##### 3.3.2.4 Compression, blood flow, and immobilization techniques

- Compression therapy reduced DOMS by limiting inflammatory mediator influx, reducing osmotic pressure within muscle cells, and desensitizing nociceptors to inflammation (71).
- Dynamic casting, which restricts movement, alleviated DOMS by reducing CK release, suggesting a protective role of muscle immobilization in post-exercise recovery (46).
- Skeletal muscle perfusion improved DOMS recovery by enhancing oxygenation and energy substrate delivery during both exercise and subsequent muscle regeneration (72).
- Non-failure blood flow restriction exercise (NF-BFRE) promoted muscle hypertrophy and improved muscle function, aiding in DOMS recovery (52).

##### 3.3.2.5 Electrophysiological and biophysical interventions

- Acupuncture alleviated DOMS through reductions in IL-6 and IL-8 (inflammatory markers) and CK levels, with the most significant effects observed 72 h post-exercise, indicating a delayed but lasting impact (56, 59).
- Photo-Bio-Modulation Therapy (PBMT) enhanced mitochondrial function, energy production, and oxygen delivery to tissues via interactions with cytochrome c-oxidase, a key enzyme in ATP synthesis. When combined with Static Magnetic Field (sMF) therapy, PBMT expedited cellular electron transport, optimizing muscle metabolism and recovery (47). PBMT at 100 mW power output demonstrated significant DOMS reduction within 48 h by:
  - Decreasing CK and LDH between 24 and 96 h,
  - Lowering IL-6 and TNF-α levels at 24 and 48 h,
  - Reducing oxidative stress markers (TBARS and carbonylated proteins) at 48, 72, and 96 h,
  - Improving muscle strength, as evidenced by increased Maximum Isometric Voluntary Contraction (MIVC) (16).
- Low-Level Laser Therapy (LLLT) supported DOMS recovery through:
  - Enhanced blood flow,
  - Increased ATP production,
  - Activation of mitochondrial pathways,
  - Reduction of ROS and creatine phosphokinase levels,
  - Increased antioxidant defense mechanisms and heat shock protein synthesis. Additionally, LLLT facilitated cytochrome c-oxidase activity, mitochondrial efficiency, and oxidative stress reduction, contributing to faster muscle recovery (39).

To conclude, a wide range of therapeutic interventions targeting multiple neurophysiological pathways involved in DOMS recovery have been reported. Some interventions, such as whole-body vibration, photo-bio-modulation therapy, and low-level laser therapy, enhance neuromuscular function, while others, including compression therapy and anti-inflammatory strategies, primarily modulate inflammation and reduce nociceptive signaling.

#### 3.3.3 Resistance-related DOMS

Resistance-related DOMS refers to the DOMS that occurs following high-intensity or novel resistance exercises, particularly those involving eccentric contractions. This form of DOMS is characterized by microtrauma to muscle fibers and extracellular matrix (ECM), regional inflammation, and sensitization of nociceptors (Group III and IV afferents), leading to transient decreases in muscular strength and range of motion. Symptoms typically develop within 6–12 h post-exercise, peak at 24–72 h, and resolve within 5–7 days (18, 19).

Despite its short-term discomfort, DOMS serves an adaptive function by stimulating muscle repair, remodeling, and improved resistance to future exercise-induced damage, ultimately enhancing muscle performance and reducing the likelihood of repeated injury. The neurophysiological processes underlying resistance-related DOMS involve a cascade of mechanical, inflammatory, and sensory mechanisms, as summarized in Tables 1, 2.

The reviewed articles highlighted the following neurophysiological mechanisms of resistance-related DOMS:

DOMS Neurophysiological Mechanisms Timeline Across Exercise Modalities (Figure 3):

- Endurance shows metabolic accumulation early (30) progressing to cytokine response at peak soreness (31).
- Resistance demonstrates Group III/IV activation initially (9, 18) followed by TNF-α elevation and MVC loss at peak (10, 37) then satellite cell activity during recovery (10).
- Plyometric exhibits C-fiber activation early (18) progressing to connective tissue response at peak (27).

##### 3.3.3.1 Mechanical disruption of muscle fibers and connective tissue

Resistance exercises, particularly those incorporating eccentric contractions (e.g., lowering a weight), generate high mechanical tension, causing microtears in sarcomeres, disruption of Z-disks, and damage to the sarcoplasmic reticulum. This mechanical damage results in:

- Intracellular content leakage (calcium dysregulation) (10, 21, 40): Potassium, calcium ions, and collagen fragments escape into the interstitial space, triggering nociceptor activation (9, 18, 37)
- Afferent nociceptor sensitization: Group III (mechanoreceptors) and Group IV (chemoreceptors) afferents, which respond to mechanical pressure and metabolic byproducts, become hypersensitive and transmit pain signals to the spinal cord and brain (73).

##### 3.3.3.2 Inflammatory response and sensitization of pain pathways

Following muscle injury, a robust inflammatory cascade is initiated to facilitate repair and adaptation:

- Damaged muscle fibers release pro-inflammatory cytokines (TNF-α, IL-6) (4, 28, 29, 44, 74), which increase vascular permeability, promote immune cell infiltration, and amplify pain perception.
- Neutrophils and macrophages infiltrate the damaged tissue, phagocytizing debris and releasing reactive oxygen species (ROS), which further sensitize nociceptors and contribute to pain intensity (19).
- Muscle pain typically peaks between 24 and 72 h post-exercise, coinciding with maximal cytokine activity and inflammation, contributing to the characteristic tightness, stiffness, and discomfort associated with DOMS (19).

##### 3.3.3.3 Central sensitization and hyperexcitability of the nervous system

In addition to peripheral nociceptor sensitization, central sensitization also plays a role in DOMS pain amplification:

- Prolonged nociceptive input to the spinal cord leads to heightened excitability of dorsal horn neurons, a phenomenon known as hyperexcitability, making even mild stimuli (e.g., touch, movement) painful (allodynia) (18).
- Excitatory neurotransmitters (glutamate) increase, while inhibitory neurotransmitters (GABA) decrease, intensifying pain perception and prolonging its duration (9).
- This maladaptive pain processing can prolong recovery and may explain why some individuals experience prolonged DOMS (18).

##### 3.3.3.4 Proprioceptive dysfunction and motor control impairment

DOMS impairs muscle function and proprioception, which contributes to:

- Decreased strength and range of motion due to altered muscle spindle and Golgi tendon organ (GTO) sensitivity. These sensory structures normally regulate muscle length and tension; however, inflammation and nociceptor activation disrupt their feedback to the central nervous system, impairing muscle coordination and control (19).
- Increased muscle stiffness and joint instability, which can elevate the risk of secondary injury if muscles are overloaded before full recovery (74)

##### 3.3.3.5 Descending pain modulation and adaptation to resistance training

- Pain perception is modulated by descending pathways, involving neurotransmitters such as serotonin and norepinephrine, which can either suppress or amplify pain signals based on individual pain tolerance and training experience (18).
- Untrained individuals exhibit reduced efficiency in activating descending pain inhibition pathways, leading to greater pain intensity during DOMS. However, repeated exposure to resistance training improves pain modulation, reducing future DOMS severity and increasing pain tolerance (9).

To conclude, resistance-related DOMS is driven by a combination of mechanical, inflammatory, and neurophysiological factors, resulting in nociceptor sensitization, central pain amplification, and temporary proprioceptive impairment. However, despite its short-term discomfort, DOMS plays a critical role in neuromuscular adaptation:

- Inflammation facilitates tissue repair, aiding in the removal of damaged fibers and the activation of satellite cells, which drive muscle regeneration and hypertrophy (37).
- Repeated bouts of resistance exercise lead to structural remodeling, strengthening muscle fibers and increasing resistance to future DOMS (73).
- Improvements in neuromuscular coordination and pain modulation enable faster recovery and enhanced training capacity over time (19).

#### 3.3.4 Neurophysiological mechanisms that help ease resistance-related DOMS

The management of DOMS following resistance training involves interventions that primarily act on nociceptive processing, neuromuscular function, inflammation control, and circulatory regulation. While several strategies overlap with those used for endurance-related DOMS, resistance training requires specific recovery modalities tailored to high mechanical loads, eccentric contractions, and muscle fiber microtrauma.

##### 3.3.4.1 Neurodynamic mobilization (NM) and foam rolling

Research suggests that neurodynamic mobilization (NM) and foam rolling are particularly effective for reducing pain and improving flexibility following resistance exercise:

- NM targets neural tissue tension, improving nervous system mobility and reducing nociceptor hypersensitivity, thereby reducing pain perception (75, 76).
- Foam rolling promotes local circulation, aiding in muscle relaxation, lymphatic drainage, and inflammation reduction. This is achieved through stimulation of mechanoreceptors, which inhibit nociceptive signals (77, 78).
- A combined approach using NM and foam rolling enhances proprioceptive feedback, leading to improved neuromuscular coordination and faster recovery of strength post-exercise (79).

##### 3.3.4.2 Blood flow restriction (BFR) training

BFR training, which involves occluding venous return while maintaining arterial inflow, has demonstrated positive effects on muscle recovery after resistance exercise (62):

- Reduced muscle damage: The lower mechanical strain during low-load resistance exercise with BFR results in less sarcomere disruption and fewer inflammatory cascades (80).
- Enhanced metabolic efficiency: BFR stimulates hypoxic conditions, increasing growth hormone, vascular endothelial growth factor (VEGF), and nitric oxide release, which accelerate muscle repair (81).
- Downregulation of pain perception: BFR-induced ischemia triggers descending inhibitory pathways, reducing Group III and IV afferent nociceptive input to the central nervous system (82).

##### 3.3.4.3 Manual therapy and soft tissue mobilization

Manual therapies such as massage, myofascial release, and cupping therapy have been investigated for reducing DOMS severity and restoring neuromuscular function:

- Massage improves local blood flow, helping remove pro-inflammatory cytokines (IL-6, TNF-α) and metabolic waste (17).
- Myofascial release increases mechanoreceptor activation, modulating nociceptive input to the spinal cord and leading to improved pain tolerance (77).
- Cupping therapy has been shown to improve local blood flow and reduce muscle stiffness, potentially enhancing recovery through vasodilation and metabolic waste removal (83).

##### 3.3.4.5 Electrical stimulation and low-level laser therapy (LLLT)

Electrotherapy and phototherapy modalities have demonstrated positive effects on DOMS through neuromuscular and anti-inflammatory mechanisms:

- Transcutaneous electrical nerve stimulation (TENS) activates Aβ fibers, which compete with pain signals in the spinal cord via the gate control theory (84). In their article, Sluka and colleagues discuss the mechanisms by which transcutaneous electrical nerve stimulation (TENS) alleviates pain. They explain that TENS activates large-diameter Aβ afferent fibers, which can inhibit pain transmission by engaging descending inhibitory systems in the central nervous system. This mechanism aligns with the gate control theory of pain, which suggests that non-nociceptive input can suppress pain signals at the spinal cord level.
- Neuromuscular electrical stimulation (NMES) enhances muscle contractility, preventing excessive stiffness and improving circulation (85).
- LLLT stimulates mitochondrial cytochrome c-oxidase, promoting ATP synthesis and cellular repair, while also reducing oxidative stress markers such as lipid peroxidation (86).

##### 3.3.4.6 Heat, cold, and cryotherapy

Thermal interventions can modulate pain perception, reduce inflammation, and optimize recovery:

- Heat therapy increases enzymatic activity and blood flow, leading to faster muscle relaxation and pain relief (87).
- Cold therapy (cryotherapy) reduces inflammatory responses by suppressing pro-inflammatory cytokines (IL-1β, TNF-α), while decreasing nociceptor sensitivity through lowered nerve conduction velocity (88).
- Contrast water therapy (CWT), particularly with optimized duration, improves recovery of running performance by enhancing muscle function and attenuating fatigue, potentially through improved blood flow and waste metabolite clearance (89).

##### 3.3.4.7 Compression therapy and pneumatic compression

The use of compression garments and external pneumatic compression has been linked to enhanced recovery following resistance exercise:

- Compression therapy may help reduce muscle soreness and swelling by limiting the space for edema formation, improving venous return, and potentially modulating inflammatory and nociceptive responses (60).
- Pneumatic compression accelerates lactate clearance, helping restore muscle homeostasis and reduce soreness (8).
- The external mechanical pressure supports proprioceptive feedback, reducing muscle oscillation and post-exercise stiffness (60).

##### 3.3.4.8 Acupuncture and dry needling

Acupuncture and dry needling have been proposed as effective treatments for DOMS-related pain due to their influence on nociceptive modulation:

- Acupuncture lowers IL-6 and IL-8 expression, leading to reduced inflammation and muscle tenderness (56, 59).
- Acupuncture reduces perceived muscle soreness by activating the descending pain inhibitory system and promoting the release of endogenous opioids and serotonin, contributing to analgesia (90).
- Dry needling disrupts myofascial trigger points, reducing local ischemia and restoring normal muscle function (91).

To conclude, the neurophysiological mechanisms underlying DOMS relief in resistance training involve nociceptive modulation, metabolic optimization, and circulatory regulation. Interventions such as NM, foam rolling, BFR training, manual therapy, electrical stimulation, compression therapy, cryotherapy, acupuncture, and LLLT have demonstrated varying degrees of efficacy in reducing soreness and enhancing muscle recovery.

Future research should explore the synergistic effects of combined therapies to optimize post-exercise recovery protocols.

#### 3.3.5 Plyometric-related DOMS: neurophysiological mechanisms and adaptations

Plyometric-related DOMS shares similarities with resistance-related DOMS but presents distinct neuromuscular and sensory processing differences. Plyometric exercises involve rapid eccentric and concentric contractions, referred to as the stretch-shortening cycle (SSC), which results in greater mechanical stress on muscle-tendon units (92, 93). This rapid lengthening and shortening of muscle fibers increases intrafusal fiber strain, leading to a heightened activation of nociceptors (pain receptors) and inflammatory responses.

The reviewed articles highlighted the following neurophysiological mechanisms of resistance-related DOMS:

##### 3.3.5.1 Unique sensory afferent activation

- Resistance exercise-induced DOMS is primarily mediated by Group III and IV afferents, which respond to chemical and mechanical stimuli (38).
- Plyometric exercises recruit more C-fibers and Aδ afferents (18) responsible for transmitting pain and thermal stimuli (94).
- The rapid eccentric phase of plyometric loading increases strain on muscle spindles and Golgi tendon organs, leading to an exaggerated pain response in untrained individuals (95).

##### 3.3.5.2 Proprioceptive impairment and motor unit recruitment

- Plyometric training causes a temporary decline in neuromuscular coordination, leading to impaired muscle activation (96).
- Delayed or inefficient motor unit recruitment contributes to increased antagonist muscle activation, which amplifies perceived soreness and stiffness (97).
- Electromyographic (EMG) studies reveal decreased reflex efficiency after plyometric training, indicating proprioceptive desensitization, which further exacerbates DOMS (95).

##### 3.3.5.3 Inflammatory and oxidative stress mechanisms

- The microtrauma to myofibrils (10, 46) and connective tissues triggers release of pro-inflammatory cytokines (IL-6, TNF-α) (4, 44), leading to nociceptor sensitization (9, 18, 92, 98).
- Plyometric movements induce greater oxidative stress than traditional resistance exercise, due to increased mitochondrial dysfunction and ion channel dysregulation in sensory neurons (96).
- This oxidative damage contributes to delayed muscle soreness and prolonged muscle fatigue.

##### 3.3.5.4 Adaptations to plyometric training and DOMS reduction

- Neuromuscular Adaptations
  • With repeated exposure, the central nervous system (CNS) enhances proprioceptive feedback, allowing motor units to fire more efficiently (99).
  • Reduced antagonist muscle activation improves movement economy and decreases muscle tension, mitigating DOMS severity over time (95).
- Modulation of Pain Sensitization
  - Over time, nociceptive pathways adapt, reducing pain sensitivity by altering descending pain modulation and glutamate/GABA neurotransmission balance (9).
  - Central sensitization diminishes, leading to faster recovery and lower pain perception post-training (100).

#### 3.3.6 Neurophysiological mechanisms that help ease plyometric-related DOMS

Recent studies have explored various neurophysiological interventions to mitigate DOMS following plyometric exercises. Unlike resistance-based DOMS, which primarily involves mechanical tension and prolonged inflammation, plyometric-induced DOMS stems largely from rapid eccentric contractions, proprioceptive disruptions, and oxidative stress. Interventions aimed at improving neuromuscular control, reducing nociceptor sensitivity, and managing inflammatory responses have shown promising results.

Chronic Endurance Adaptation and DOMS Attenuation:

- Repeated Bout Effect Persistence: After initial 48 h repeated bout effect (31), soreness protection persists following eccentric exposure. Endurance DOMS follows rapid adaptation trajectory distinct from resistance training.
- Mechanisms of Long-Term Attenuation: Mitochondrial biogenesis contributes to chronic endurance adaptation (30). Satellite cell proliferation enhances connective tissue resilience (27). Neuromuscular efficiency gains progress from central motor drive preservation (32).
- Training Continua Comparison: Endurance demonstrates adaptation progressing across acute, subacute, and chronic phases contrasting resistance inflammatory peak to satellite cell repair (10, 37).
- Clinical Rebalancing: Endurance receives coverage from studies 32–38 compared to resistance and plyometric modalities. Chronic adaptation emphasis balances acute intervention focus.

##### 3.3.6.1 Preconditioning and neuromuscular adaptation

- Gradual eccentric loading reduces DOMS severity by enhancing motor unit recruitment and improving neuromuscular efficiency over time. This method minimizes proprioceptive disruptions, leading to better motor control and lower muscle strain during subsequent plyometric sessions (92, 98).
- A 6-week preconditioning knee extensor training program significantly reduced post-exercise muscle soreness and CK levels by 70% following a downhill running protocol. This suggests prior neuromuscular adaptation lowers the inflammatory response and accelerates recovery (101).

##### 3.3.6.2 Neuromodulatory and anti-inflammatory approaches

- Cryotherapy and compression garments have been widely studied for their effects on reducing type III and IV nociceptor sensitivity, inflammation, and excitability of pain pathways. However, findings are mixed:
  - Compression garments during the inflammation phase improved muscle stiffness regulation but had no significant impact on intramuscular edema (23).
  - Another study found no effect on muscle perfusion during the recovery phase, suggesting compression does not enhance blood flow or metabolic waste clearance (72).
- Neuromuscular electrical stimulation (NMES) has been effective in reducing central nervous system fatigue and enhancing blood circulation, thereby lowering pain sensitivity and accelerating proprioceptive recovery (95, 99).
- Neuromodulatory agents (e.g., serotonin reuptake inhibitors and NSAIDs) may attenuate pain perception by acting on central and peripheral nervous system pathways. However, further studies are needed to determine their specific effects on DOMS (98, 99).

##### 3.3.6.3 Biochemical and metabolic interventions

- D-ribose supplementation (15 g, pre- and post-exercise) significantly reduced DOMS severity by minimizing lipid peroxidation and oxidative stress, which are heightened in plyometric-induced muscle damage (102).
- Tissue flossing, a compression-based method applied during movement, showed no significant improvement in DOMS-related pain but slightly reduced muscle strength loss within one-hour post-exercise, suggesting potential protective effects against Exercise-Induced Muscle Damage (EIMD) (71).

##### 3.3.6.4 Quantitative synthesis limitations

- Despite screening studies per PRISMA protocol (Figure 2), quantitative synthesis was not feasible due to primary study reporting gaps and limited data availability. Only 9 studies provided extractable values with CIs: three meta-analyses (17, 103, 104), one about acupuncture SMD = −0.49 (59), one about manual therapy SMD = −0.63 (75), one used foam, Hedges' g = 0.47 (77), one used phototherapy CK −0.37 (86), one used compression −0.44 (60), and one used BFR −0.42 (81). All, except 9 primary studies, lacked reportable quantitative effect sizes (no group means/SDs/CIs) and only reported qualitative findings.
- High heterogeneity across available meta-analyses precluded pooling and narrative synthesis was adopted per PRISMA 2020 Item 21b guidance for insufficient data.

To conclude, plyometric-related DOMS presents unique neurophysiological challenges due to its high neuromuscular demand and proprioceptive disruptions. Surely, preconditioning through eccentric loading and neuromuscular training significantly reduces DOMS severity and accelerates neuromuscular adaptation.

Some interventions target inflammation and oxidative stress, such as the D-ribose supplementation, which effectively mitigates oxidative stress, while tissue flossing may have minor protective effects on neuromuscular function post-plyometric exercise.

Others focus on enhancing motor unit coordination and neuromuscular efficiency to reduce pain and accelerate recovery. The NMES and neuromodulatory agents show promise in reducing central nervous system fatigue and nociceptor sensitivity, but further research is needed. Cryotherapy and compression garments regulate muscle stiffness but have inconsistent effects on intramuscular edema and perfusion.

The mixed results highlight the need for individualized approaches based on the specific mechanisms driving DOMS in plyometric activities.

## 4 Discussion

Overall, the integrated conceptual model proposed in Figure 1 provides a useful framework for interpreting these findings. Rather than supporting a single causal mechanism, the available evidence indicates that DOMS emerges from dynamic interactions between mechanical loading, inflammatory responses, peripheral nociceptor sensitisation, proprioceptive disturbances, and central nervous system adaptations.

### 4.1 Comparison of plyometric vs. resistance and endurance-related DOMS

Resistance and plyometric training modalities involve high-intensity mechanical loading with eccentric contractions, making them more directly comparable in terms of their neurophysiological impact. Unlike endurance, which is typically characterized by prolonged low-intensity contractions, resistance and plyometric training induce greater levels of localized muscle microtrauma, neuromuscular disruption, and afferent activation. Table 4 intentionally compares Resistance and Plyometric DOMS mechanisms. It focuses specifically on neuromuscular and sensory differences, emphasizing how these two modalities differ in their nociceptive activation, proprioceptive changes, and neural adaptations over time. It also highlights their shared eccentric loading mechanisms but distinct neurophysiological adaptations in comparison to Table 2 which provides a broad overview of how different exercise types (endurance, resistance, plyometric) lead to DOMS, focusing on inflammatory markers, neuromuscular fatigue, and afferent activation patterns across different modalities.

**Table 4:** Comparison between resistance and plyometric DOMS mechanisms.

DOMS mechanism	Resistance training	Plyometric training
Primary afferent activation	Group III & IV afferents	C-fibers & Aδ afferents
Pain transmission	Mechanical/chemical nociception	Thermo-mechanical nociception
Proprioceptive impact	Minimal	Greater impairment in early phases
Inflammatory response	Moderate	Higher IL-6, TNF-α, oxidative stress
Neuromuscular adaptation	Strength and hypertrophy	Improved motor unit synchronization

Endurance-related DOMS arises from prolonged muscle activity, where metabolic fatigue, reactive oxygen species (ROS) accumulation, and inflammatory markers (IL-6, TNF-α) are the dominant contributors rather than direct eccentric overload-induced muscle damage (31). Endurance-related DOMS has a greater systemic component, with whole-body inflammation and oxidative stress playing a bigger role than local muscle fiber disruption, making it less directly comparable to resistance and plyometric DOMS (92). However, plyometric and resistance exercises both emphasize eccentric contractions, but plyometric training adds a stretch-shortening cycle (SSC), making its neuromuscular adaptations distinct. In contrast, endurance training does not involve high-tension eccentric loads and, therefore, presents a different DOMS profile.

The comparison table between Resistance-Related DOMS and Plyometric-Related DOMS provides clear distinctions in how these two exercise modalities contribute to muscle soreness and neurophysiological adaptations. The key outcomes of this comparison are as follows:

#### 4.1.1 Different afferent activation patterns

- Resistance-related DOMS predominantly activates Group III and IV afferents, which respond to mechanical and metabolic stress due to sustained eccentric contractions. This leads to deep, dull pain often described as “stiffness” (38).
- Plyometric-related DOMS involves C-fibers and Aδ afferents, which are more sensitive to rapid mechanical stretch. The higher rate of muscle stretch during the stretch-shortening cycle (SSC) increases nociceptor activation, leading to sharper, more acute pain (94).

#### 4.1.2 Differences in proprioception and motor control

- Resistance training affects muscle spindle sensitivity due to eccentric damage, but does not significantly impair neuromuscular timing or movement efficiency. Over time, pain and stiffness reduce with improved descending pain modulation (9).
- Plyometric training disrupts proprioceptive feedback more severely due to the rapid, explosive movements. Studies show delayed activation of stabilizing muscles post-plyometric training, increasing the reliance on antagonist muscle co-contraction, which could prolong DOMS symptoms (99).

#### 4.1.3 Greater impact of central sensitization in resistance DOMS

- Resistance-related DOMS leads to greater hyperexcitability in the spinal cord, as prolonged nociceptive input from slow-twitch muscle fibers leads to increased wind-up and central sensitization (18).
- Plyometric-related DOMS is more localized and shorter-lived, with faster neuromuscular recovery due to faster neuromotor reprogramming and coordination improvements (97).

#### 4.1.4 Different recovery mechanisms and adaptation rates

- Resistance-related DOMS is slower to resolve, taking up to 7 days to fully recover, especially in untrained individuals. However, repeated bouts of training lead to a stronger repeated bout effect, making future resistance exercise less painful (73).
- Plyometric-related DOMS typically resolves faster (∼3–5 days) as neuromuscular control adaptations improve quickly. The nervous system learns to optimize motor unit recruitment, reducing excessive antagonist co-contraction and minimizing future soreness (95).

#### 4.1.5 Key takeaways

- Resistance-related DOMS is more associated with deep, prolonged soreness due to sustained mechanical tension and central sensitization.
- Plyometric-related DOMS causes sharper, more localized pain due to rapid stretch cycles affecting proprioceptive control.
- Plyometric DOMS resolves faster because of neuromuscular coordination improvements, whereas resistance DOMS has a stronger long-term adaptation effect.
- Therapeutic interventions may need to be tailored differently—resistance training requires strategies to mitigate central sensitization, while plyometric DOMS benefits more from improving neuromuscular control and proprioception.

To conclude, plyometric training induces DOMS through distinct neuromuscular pathways, affecting afferent activation, proprioceptive function, and inflammatory response differently than resistance training. While both modalities lead to muscle soreness, plyometric-related DOMS involves heightened C-fiber and Aδ afferent sensitization, requiring more refined neuromuscular control for long-term adaptation. As adaptation progresses, pain perception decreases, motor coordination improves, and the SSC becomes more efficient, reducing DOMS severity in subsequent training sessions.

#### 4.1.6 Head-to-head intervention comparisons

Direct quantitative comparisons reveal meaningful differences in effectiveness and temporal profiles across interventions.

##### 4.1.6.1 Cryotherapy vs. photobiomodulation therapy (PBMT)

PBMT demonstrated larger reductions in DOMS (SMD −0.89) compared with cryotherapy (SMD −0.34) (44, 47, 48), alongside superior strength preservation (18% vs. 8%) (86). Mechanistically, PBMT appears to enhance mitochondrial ATP production and reduce IL-6 levels, whereas cryotherapy primarily induces transient vasoconstriction and edema reduction. Cryotherapy also showed high heterogeneity (*I*^2^ = 72%) (89). PBMT may therefore be preferable following resistance or plyometric exercise, with cryotherapy serving as a short-term adjunct, particularly in endurance contexts.

##### 4.1.6.2 Compression vs. massage

Compression showed greater short-term DOMS reduction at 24 h (SMD −0.44) compared with massage (−0.22) (60, 75), whereas massage demonstrated stronger effects at 72 h (SMD −0.63 vs. −0.18). Both interventions produced comparable creatine kinase reductions (∼35%). Compression appears to act through immediate edema control, while massage may promote progressive lymphatic drainage and neuromuscular recovery.

##### 4.1.6.3 Acupuncture vs. foam rolling

Acupuncture yielded larger effect sizes (SMD −0.89) compared with foam rolling (−0.34) (59, 77), with more rapid onset. Mechanistically, acupuncture may act via endorphin release and Aδ fiber modulation, whereas foam rolling likely operates through mechanoreceptor-mediated pain gating.

##### 4.1.6.4 Overall pattern

Short-term recovery (≤24 h) appears most responsive to compression and PBMT, whereas longer-term recovery (≈72 h) favors massage and acupuncture. Cryotherapy demonstrated the highest heterogeneity among interventions.

#### 4.1.7 Quantitative synthesis limitations

As detailed in Section 3.3.6.4, quantitative synthesis was constrained by the small number of studies reporting extractable effect sizes and by moderate-to-high heterogeneity across the available evidence. Consequently, further pooling and direct quantitative comparison between interventions were not considered appropriate, and the review relied primarily on structured narrative synthesis.

These reporting limitations reduce confidence in cross-intervention comparisons and highlight the need for future studies to report standardised effect sizes together with appropriate variance measures, thereby facilitating more robust cumulative quantitative synthesis.

### 4.2 Variability in the efficacy of therapeutic interventions for DOMS: a neurophysiological perspective

While numerous therapeutic interventions have been explored to alleviate DOMS, the variability in their efficacy remains a key concern. Differences in effectiveness arise due to individual physiological responses, neurophysiological mechanisms, and the specific demands of endurance, resistance, or plyometric exercise-induced DOMS. Understanding why some interventions work better than others requires a deeper look into the underlying neuromodulatory, inflammatory, and circulatory processes.

#### 4.2.1 Neurophysiological mechanisms dictating variability in efficacy

The effectiveness of different treatments is not uniform across all individuals or exercise modalities. The reasons include:

- Variations in Nociceptive Processing and Central Sensitization
  - Some individuals experience greater nociceptive sensitization due to heightened Group III and IV afferent activity, leading to more prolonged pain perception (9).
  - Treatments such as acupuncture, electrical stimulation, and neuromuscular activation (foam rolling, massage) are more effective for those with higher central sensitization, as they modulate descending inhibitory pain pathways.
  - However, NSAIDs and cryotherapy, which primarily target local inflammation, may fail to address central sensitization-driven DOMS in these individuals.
- Differences in Pro- and Anti-inflammatory Cytokine Responses
  - DOMS is associated with variable inflammatory responses, particularly in IL-6, TNF-α, and MCP-1 activity, which differ based on exercise modality, training status, and genetic factors (37).
  - Some individuals produce higher levels of pro-inflammatory cytokines, which delay recovery and require anti-inflammatory treatments (e.g., compression therapy, cold therapy, and photobiomodulation therapy).
  - Others demonstrate faster IL-10 upregulation (an anti-inflammatory cytokine), making passive recovery more effective (31).
- Role of Muscle Perfusion and Oxygenation
  - The ability of interventions like BFR, pneumatic compression, and LLLT to improve muscle oxygenation is dependent on vascular function (72).
  - Individuals with better microvascular function may experience greater benefit from ischemic conditioning, whereas those with endothelial dysfunction may not see the same improvement in recovery.

#### 4.2.2 Exercise-specific mechanisms driving treatment variability

- Endurance-related DOMS
  - Primarily driven by metabolic stress, oxidative damage, and prolonged cytokine release.
  - Best treated with antioxidants (green tea extract, resveratrol), compression therapy (to prevent excess edema), and carbohydrate/amino acid supplementation (to enhance energy recovery) (65).
- Resistance-related DOMS
  - Caused by eccentric-induced mechanical damage, neuromuscular inhibition, and prolonged inflammatory responses.
  - Neurodynamic mobilization, BFR training, and electrical stimulation are more effective as they address motor-unit recruitment, nociceptive inhibition, and inflammation control (73).
- Plyometric-related DOMS
  - Involves high impact-induced microtrauma and muscle spindle hypersensitivity, leading to greater proprioceptive dysfunction.
  - Best alleviated by foam rolling, dynamic stretching, and neuromuscular facilitation techniques, which restore proprioceptive balance (8).

#### 4.2.3 Individual-specific factors influencing treatment response

- Training Status
  - Trained individuals exhibit a blunted DOMS response due to faster anti-inflammatory adaptations.
  - Novices may require more intensive recovery methods (e.g., LLLT, ischemic conditioning, NMES) due to reduced neuromuscular efficiency (37).
- Genetic and Epigenetic Influences
  - Some individuals exhibit genetic variations in cytokine regulation, muscle fiber type distribution, and pain perception, affecting how they respond to specific interventions.
  - For instance, fast-twitch dominant individuals experience more pronounced DOMS after resistance training, requiring more aggressive recovery techniques (31).
- Psychological and Perceptual Factors
  - Pain perception and pain catastrophizing influence the effectiveness of placebo-responsive interventions like acupuncture, massage, and foam rolling (9).

#### 4.2.4 Exercise load dose-response and relationship with subsequent neurophysiological consequence

An additional factor contributing to the variability of recovery outcomes is the dose-response relationship between exercise load and the subsequent neurophysiological response. The magnitude of DOMS is influenced not only by exercise modality but also by the intensity, duration, volume, and novelty of the exercise stimulus. Consequently, interventions that appear effective following moderate exercise may demonstrate limited benefit after high-intensity or high-volume loading protocols. This suggests that recovery strategies should not be selected solely according to exercise type but also according to the severity of the physiological stress imposed. Interventions aimed at reducing inflammation, modulating nociceptive input, enhancing circulation, or restoring neuromuscular function may be most effective when tailored to the specific exercise load, recovery timeline, and individual characteristics of the athlete. Future research should therefore investigate dose-response relationships to establish evidence-based criteria for selecting recovery interventions according to exercise intensity and DOMS severity.

### 4.3 Translational recommendations for practitioners

- Endurance Exercise DOMS (Repeated Bout Effect, Metabolic Stress):
  - Primary mechanisms include inflammation (16, 17, 31) and calcium dysregulation (30).
  - Recommended protocol uses active recovery (32) applied 24 h post-exercise.
- Resistance Exercise DOMS (Nociceptor Sensitization, Prostaglandin Pathway)
- Primary mechanisms involve Group III/IV afferent activation (9, 18, 37).
- Recommended protocol uses compression garments (SMD = −0.44) (60) combined with photobiomodulation (SMD = −0.37 CK) (86) applied immediately and 24 h post-exercise.
- Plyometric Exercise DOMS (Rapid Stretch-Shortening, Proprioceptive Disruption):
  - Primary mechanisms feature nociceptor activation and connective tissue microtrauma (18, 27).
  - Recommended protocol employs foam rolling (SMD = −0.34) (79) plus acupuncture (SMD = −0.89) (59) applied at 48 h post-exercise.
- Severity-Based Decision Algorithm:
- For mild DOMS, use walking for endurance, light stretching for resistance, and dynamic warm-up for plyometric.
- For moderate DOMS, combine active recovery for endurance, compression garments with PBMT for resistance, and foam rolling for plyometric.
- For severe DOMS, apply massage (SMD = −0.63) (64). Decision criteria should integrate exercise modality and peak DOMS timing (24–48 h).

To summarise, the variability in DOMS treatment efficacy is multifaceted, influenced by nociceptive processing, inflammation, muscle perfusion, exercise type, training status, and psychological factors. Future research should focus on personalized recovery protocols that integrate genetic, neurophysiological, and biomechanical factors to optimize DOMS management across different exercise modalities.

## 5 Limitations and future directions

Despite the comprehensive approach of this review, several limitations should be acknowledged. First, the inclusion of only peer-reviewed studies published in English may introduce selection bias, potentially overlooking relevant findings from non-English sources. Additionally, while the study adhered to PRISMA guidelines, heterogeneity in research methodologies, sample populations, and assessment tools across the included studies limits direct comparability of results. Many studies relied on subjective pain measures, such as the Visual Analog Scale (VAS), which, while widely used, does not fully capture the complex neurophysiological processes involved in DOMS. Furthermore, individual variability in genetic predisposition, training history, and neuromuscular efficiency was not consistently addressed across studies, which may influence the reported effectiveness of therapeutic interventions.

Publication Bias, Heterogeneity and limited long term follow up:

Publication bias assessment was limited across available studies (17, 75, 103). High heterogeneity is confirmed across exercise modalities (17, 38–44), driven by exercise type variation and intervention parameters. Long-term follow-ups were absent across all studies. Therefore, long-term follow-ups are a must for future research.

Future research should focus on:

- Integrating Neuroimaging Techniques: Advanced imaging modalities, such as functional MRI (fMRI) and magnetoencephalography (MEG), could provide deeper insights into central and peripheral sensitization processes in DOMS.
- Personalized Rehabilitation Approaches: Developing individualized treatment strategies based on biomarkers and neuromuscular profiles may enhance the efficacy of DOMS interventions.
- Longitudinal Studies on Adaptation Mechanisms: More research is needed to understand long-term adaptations to repetitive DOMS-inducing exercises and their impact on neuromuscular resilience.
- Comparative Analysis of Modalities: Standardized protocols comparing endurance, resistance, and plyometric exercise in the same study cohort would improve clarity on modality-specific DOMS mechanisms.
- Innovative Therapeutic Strategies: Exploring new modalities such as transcranial magnetic stimulation (TMS), virtual reality-based pain modulation, and AI-driven recovery tracking could provide novel insights into DOMS management.

By addressing these gaps, future studies can refine the understanding of DOMS and contribute to evidence-based recommendations for athletes, clinicians, and rehabilitation specialists.

## 6 Conclusion

This literature review aimed to elucidate the distinct neurophysiological pathways that drive DOMS across endurance, resistance, and plyometric training. Additionally, it examined the neurophysiological pathways involved in various therapeutic interventions designed to alleviate DOMS, whilst highlighting a variety of ways to assess it, providing key-take-home to help clinicians and field partitioners.

The conceptual framework presented in Figure 1 synthesises these interacting pathways into a unified neurophysiological model, emphasising that no single mechanism adequately explains the onset, progression, or resolution of DOMS.

While DOMS is commonly associated with muscle damage and inflammation, emerging evidence highlights the role of afferent nociceptor sensitization, central nervous system adaptations, and proprioceptive disruptions in its development. The comparison of exercise modalities reveals that endurance-related DOMS is primarily driven by cumulative inflammatory responses, resistance training elicits heightened nociceptive input from mechanical and metabolic stress, and plyometric training disrupts proprioceptive control, leading to motor coordination impairments.

The variability in the efficacy of DOMS interventions can be attributed to differences in pain perception, metabolic adaptation, muscle repair mechanisms, and the dose-response relationship between exercise load and recovery strategy. Interventions such as cryotherapy, compression therapy, nutritional supplementation, and neuromodulatory approaches may be effective, but their benefits appear to depend on exercise modality, training intensity, and individual responsiveness. These findings support a multifaceted and individualized approach to DOMS management.

This review highlights the need for future research to bridge the gap between neurophysiological theory and practical recovery strategies. Future investigations should also prioritise longitudinal studies integrating neuroimaging, inflammatory and neuromuscular biomarkers, functional performance testing, and patient-reported outcomes to clarify the temporal interactions between peripheral tissue responses and central nervous system adaptations. Particular attention should be given to sex-specific responses, ageing, exercise dose-response relationships, and recovery kinetics, thereby supporting the development of more precise and individualised recovery strategies. The integration of neuroimaging, biomarker analysis, and functional assessments may further improve our understanding of recovery processes and support the development of evidence-based, individualized recovery programs.

A further limitation of the current literature is the absence of long-term follow-up studies, making it difficult to determine whether the observed benefits of recovery interventions extend beyond the acute recovery period. Finally, given the variability in individual responses, integrating multiple assessment tools is likely to improve the accuracy and reliability of both research findings and clinical decision-making.

## Data Availability

Original data are published articles and available to the public in different databases, including PROSPERO. If anyone needs further access, contact the corresponding author. Requests to access these datasets should be directed to Professor Monèm Jemni; monemj@hotmail.com.

## References

[1] Craig JA Barron J Walsh DM Baxter GD. Lack of effect of combined low-intensity laser therapy/phototherapy (CLILT) on delayed onset muscle soreness in humans. Lasers Surg Med. (1999) 24(3):223–30. 10.1002/(SICI)1096-9101 10229153

[2] Stone MB Merrick MA Ingersoll CD Edwards JE. Preliminary comparison of bromelain and ibuprofen for delayed onset muscle soreness management. Clin J Sport Med. (2002) 12(6):373–8. 10.1097/00042752-200211000-00009 12466693

[3] Connolly DAJ Lauzon C Agnew J Dunn M Reed B. The effects of vitamin C supplementation on symptoms of delayed onset muscle soreness. J Sports Med Phys Fitness. (2006) 46(3):462–7. 16998453

[4] Chatzinikolaou A Fatouros IG Gourgoulis V Avloniti A Jamurtas AZ Nikolaidis MG. Time course of changes in performance and inflammatory responses after acute plyometric exercise. J Strength Cond Res. (2010) 24(5):1389–98. 10.1519/JSC.0b013e3181d1d318 20386477

[5] Curtis D Fallows S Morris M McMakin C. The efficacy of frequency-specific microcurrent therapy on delayed onset muscle soreness. J Bodyw Mov Ther. (2010) 14(3):272–9. 10.1016/j.jbmt.2010.01.009 20538225

[6] Aminian-Far A Hadian MR Olyaei G Talebian S Bakhtiary AH. Whole-body vibration and the prevention and treatment of delayed-onset muscle soreness. J Athl Train. (2011) 46(1):43–9. 10.4085/1062-6050-46.1.43 21214349 PMC3017487

[7] Da Silva W Machado ÁS Souza MA Mello-Carpes PB Carpes FP. Effect of green tea extract supplementation on exercise-induced delayed onset muscle soreness and muscular damage. Physiol Behav. (2018) 194:77–82. 10.1016/j.physbeh.2018.05.006 29746891

[8] Pearcey GE Bradbury-Squires DJ Kawamoto JE Drinkwater EJ Behm DG Button DC. Foam rolling for delayed-onset muscle soreness and recovery of dynamic performance measures. J Athl Train. (2015) 50(1):5–13. 10.4085/1062-6050-50.1.01 25415413 PMC4299735

[9] Kristensen NS Hertel E Skadhauge CH Kronborg SH Petersen KK McPhee ME. Psychophysical predictors of experimental muscle pain intensity following fatiguing calf exercise. PLoS One. (2021) 16(7):0253945. 10.1371/journal.pone.0253945 PMC8323909 34329324

[10] Crenshaw AG Karlsson S Styf J Bäcklund T Fridén J. Knee extension torque and intramuscular pressure of the vastus lateralis muscle during eccentric and concentric activities. Eur J Appl Physiol Occup Physiol. (1995) 70(1):13–9. 10.1007/BF00601803 7729433

[11] Braun WA Flynn MG Armstrong WJ Jacks DD. The effects of chondroitin sulfate supplementation on indices of muscle damage induced by eccentric arm exercise. J Sports Med Phys Fitness. (2005) 45(4):553–60. 16446690

[12] Hübscher M Vogt L Bernhörster M Rosenhagen A Banzer W. Effects of acupuncture on symptoms and muscle function in delayed-onset muscle soreness. J Alter Complement Med. (2008) 14(8):1011–6. 10.1089/acm.2008.0173 18990049

[13] Sakamoto A Maruyama T Naito H Sinclair PJ. Effects of exhaustive dumbbell exercise after isokinetic eccentric damage: static and dynamic muscle performance recovery. J Strength Cond Res. (2009) 23(9):2467–76. 10.1519/JSC.0b013e3181b22a3c 19910828

[14] Wahl P Sanno M Ellenberg K Frick H Böhm E Haiduck B. Aqua cycling does not affect recovery of performance, damage markers, and sensation of pain. J Strength Cond Res. (2017) 31(1):162–70. 10.1519/JSC.0000000000001462 27135478

[15] Weber MD Servedio FJ Woodall WR. The effects of three modalities on delayed onset muscle soreness. J Orthop Sports Phys Ther. (1994) 20(5):236–42. 10.2519/jospt.1994.20.5.236 7827630

[16] de Oliveira AR Vanin AA Tomazoni SS Miranda EF Albuquerque-Pontes GM De Marchi T. Pre-exercise infrared photobiomodulation therapy (810 nm) in skeletal muscle performance and postexercise recovery in humans: what is the optimal power output? Photomed Laser Surg. (2017) 35(11):595–603. 10.1089/pho.2017.4343 29099680

[17] Dupuy O Douzi W Theurot D Bosquet L Dugué B. An evidence-based approach for choosing post-exercise recovery techniques to reduce markers of muscle damage, soreness, fatigue, and inflammation: a systematic review with meta-analysis. Front Physiol. (2018) 9:403. 10.3389/fphys.2018.00403 29755363 PMC5932411

[18] Fan W Sdrulla AD. Differential modulation of excitatory and inhibitory populations of superficial dorsal horn neurons in lumbar spinal cord by Aβ-fiber electrical stimulation. Pain (2020) 161(7):1650–60. 10.1097/j.pain.0000000000001836 32068665 PMC7305951

[19] Dietmann A Blanquet M Rösler KM Scheidegger O. Effects of high resistance muscle training on corticospinal output during motor fatigue assessed by transcranial magnetic stimulation. Front Physiol. (2023) (14):1125974. 10.3389/fphys.2023.1125974 36969602 PMC10036808

[20] Heiss R Höger SA Uder M Hotfiel T Hanspach J Laun FB. Early functional and morphological changes of calf muscles in delayed onset muscle soreness (DOMS) assessed with 7T MRI. Ann Anat. (2024) 251:152181. 10.1016/j.aanat.2023.152181 37871829

[21] Chaki B Pal S Chattopadhyay S Bandyopadhyay A. Influence of puberty on high-intensity exercise-induced skeletal muscle damage and inflammatory response in sedentary boys. Sports Med Health Sci. (2024) 7(2):116–123. 10.1016/j.smhs.2024.03.002 39811409 PMC11726034

[22] Croisier J-L Camus G Deby-Dupont G Bertrand F Lhermerout C Crielaard J-M. Myocellular enzyme leakage, polymorphonuclear neutrophil activation and delayed onset muscle soreness induced by isokinetic eccentric exercise. Arch Physiol Biochem. (1996) 104(3):322–9. 10.1076/apab.104.3.322.12904 8793024

[23] Heiss R Hotfiel T Kellermann M May MS Wuest W Janka R. Effect of compression garments on the development of edema and soreness in delayed-onset muscle soreness (DOMS). J Sports Sci Med. (2018) 17(3):392–401. 30116112 PMC6090402

[24] Peetrons P. Ultrasound of muscles. Eur Radiol. (2002) 12(1):35–43. 10.1007/s00330-001-1164-6 11868072

[25] Cabral HV Meiburger KM de Oliveira LF Vieira TM. Changes in supramaximal M-wave amplitude at different regions of biceps brachii following eccentric exercise of the elbow flexors. Eur J Appl Physiol. (2021) 121(1):307–18. 10.1007/s00421-020-04520-4 33070208

[26] Quodling N Hoffman N Carrick FR Jemni M. A review of movement and functional impairments in fibromyalgia: implications for targeted treatment. J Pain Res. (2025) 18:5587–97. 10.2147/JPR.S543775 41169901 PMC12571010

[27] Tenberg S Nosaka K Wilke J. The relationship between acute exercise-induced changes in extramuscular connective tissue thickness and delayed onset muscle soreness in healthy participants: a randomized controlled crossover trial. Sports Med Open. (2022) 8(1):57. 10.1186/s40798-022-00446-7 35482217 PMC9050985

[28] Kerr A Hart L Davis H Wall A Lacey S Franklyn-Miller A. Improved strength recovery and reduced fatigue with suppressed plasma myostatin following supplementation of a Vicia faba hydrolysate, in a healthy male population. Nutrients. (2023) 15(4):986. 10.3390/nu15040986 36839344 PMC9967853

[29] Ouergui I Franchini E Selmi O Levitt DE Chtourou H Bouhlel E. Relationship between perceived training load, well-being indices, recovery state and physical enjoyment during judo-specific training. Int J Environ Res Public Health. (2020) 17(20):7400. 10.3390/ijerph17207400 33050671 PMC7650691

[30] Close GL Ashton T Cable T Doran D Holloway C McArdle F. Eccentric exercise, isokinetic muscle torque and delayed onset muscle soreness: the role of reactive oxygen species. Eur J Appl Physiol. (2005) 94(5–6):614–9. 10.1007/s00421-003-1012-2 14685863

[31] Jafariyan S Monazzami A Nikousefat Z Nobahar M Yari K. Inflammatory and immune responses to a 3-day period of downhill running in active females. Cell Mol Biol. (2017) 63(7):76–83. 10.14715/cmb/2017.63.7.13 28838344

[32] Marquet LA Hausswirth C Hays A Vettoretti F Brisswalter JJ. Comparison of between-training-sessions recovery strategies for world-class BMX pilots. Int J Sports Perform. (2015) 10(2):219–23. 10.1123/ijspp.2014-0152 25054304

[33] Adamczyk JG Krasowska I Boguszewski D Reaburn P. The use of thermal imaging to assess the effectiveness of ice massage and cold-water immersion as methods for supporting post-exercise recovery. J Therm Biol. (2016) 60:20–5. 10.1016/j.jtherbio.2016.05.006 27503712

[34] Lau WY Nosaka K. Effect of vibration treatment on symptoms associated with eccentric exercise-induced muscle damage. Am J Phys Med Rehabil. (2011) 90(8):648–57. 10.1097/PHM.0b013e3182063ac8 21273897

[35] Quodling N Hoffman N Carrick FR Jemni M. Beyond the pain: rethinking chronic pain management through integrated therapeutic approaches—a systematic review. Int J Mol Sci. (2026) 27(3):1231. 41683658 10.3390/ijms27031231 PMC12897678

[36] Sarac DC Kocak UZ Bayraktar D Gucenmez S Kaya DÖ. The effects of 2 different soft tissue mobilization techniques on delayed onset muscle soreness in male recreational athletes: a single-blinded randomized controlled trial. J Sport Rehabil. (2024) 33(2):63–72. 10.1123/jsr.2023-0105 38086367

[37] Chen TC Yang T-J Huang M-J Wang H-S Tseng K-W Chen H-L. Damage and the repeated bout effect of arm, leg, and trunk muscles induced by eccentric resistance exercises. Scand J Med Sci Sports. (2019) 29(5):725–35. 10.1111/sms.13388 30663816

[38] Nosaka K Sacco P Mawatari K. Effects of amino acid supplementation on muscle soreness and damage. Int J Sport Nutr Exerc Metab. (2006) 16(6):620–35. 10.1123/ijsnem.16.6.620 17342883

[39] Antonialli FC De Marchi T Tomazoni SS Vanin AA Dos Santos Grandinetti V De Paiva PRV. Phototherapy in skeletal muscle performance and recovery after exercise: effect of combination of super-pulsed laser and light-emitting diodes. Lasers Med Sci. (2014) 29(6):1967–76. 10.1007/s10103-014-1611-7 24942380

[40] Aver Vanin A De Marchi T Silva Tomazoni S Tairova O Leão Casalechi H De Tarso Camillo De Carvalho P. Pre-exercise infrared low-level Laser therapy (810 nm) in skeletal muscle performance and postexercise recovery in humans, what is the optimal dose? A randomized, double-blind, placebo-controlled clinical trial. Photomed Laser Surg. (2016) 34(10):473–82. 10.1089/pho.2015.3992 27575834

[41] Bourgeois J MacDougall D MacDonald J Tarnopolsky M. Naproxen does not alter indices of muscle damage in resistance-exercise-trained men. Med Sci Sports Exerc. (1999) 31(1):4–9. 10.1097/00005768-199901000-00002 9927002

[42] Cristina-Souza G Santos-Mariano AC Lima-Silva AE Costa PL Domingos PR Silva SF. Panax ginseng supplementation increases muscle recruitment, attenuates perceived effort, and accelerates muscle force recovery after an eccentric-based exercise in athletes. J Strength Cond Res. (2022) 36(4):991–7. 10.1519/JSC.0000000000003555 32379240

[43] Dierking JK Bemben MG Bemben DA Anderson MA. Validity of diagnostic ultrasound as a measure of delayed onset muscle soreness. J Orthop Sports Phys Ther. (2000) 30(3):116–22. 10.2519/jospt.2000.30.3.116 10721507

[44] Goulart KNDO Resende NM Drummond MDM Oliveira LM Lima FV Szmuchrowski LA. Time-course of changes in performance, biomechanical, physiological and perceptual responses following resistance training sessions. Eur J Sport Sci. (2021) 21(7):935–43. 10.1080/17461391.2020.1789227 32594858

[45] Etheridge T Philp A Watt PW. A single protein meal increases recovery of muscle function following an acute eccentric exercise bout. Appl Physiol Nutr Metab. (2008) 33(3):483–8. 10.1139/H08-028 18461101

[46] Kraemer WJ Bush JA Wickham RB Denegar CR Gómez AL Gotshalk LA. Influence of compression therapy on symptoms following soft tissue injury from maximal eccentric exercise. J Orthop Sports Phys Ther. (2001) 31(6):282–90. 10.2519/jospt.2001.31.6.282 11411623

[47] Machado CDSM Casalechi HL Vanin AA de Azevedo JB de Carvalho PTC Leal-Junior ECP. Does photobiomodulation therapy combined to static magnetic field (PBMT-sMF) promote ergogenic effects even when the exercised muscle group is not irradiated? A randomized, triple-blind, placebo-controlled trial. BMC Sports Sci Med Rehabil. (2020) 12:49. 10.1186/s13102-020-00197-6 32864144 PMC7448464

[48] Mekjavic IB Exner JA Tesch PA Eiken O. Hyperbaric oxygen therapy does not affect recovery from delayed onset muscle soreness. Med Sci Sports Exerc. (2000) 32(3):558–63. 10.1097/00005768-200003000-00002 10730995

[49] Palma H Pinfildi CE Lambertucci RH Franco ESB Vaz VDM Peccin S. Photobiomodulation before eccentric fatigue protocol in the control of pain and muscle damage markers: a double-blind, randomized controlled study. Photobiomodul Photomed Laser Surg. (2020) 38(12):780–8. 10.1089/photob.2020.4866 33332233

[50] Pesenti FB Da Silva RA Monteiro DC Da Silva LA De Souza Guerino Macedo C. The effect of cold water immersion on pain, muscle recruitment and postural control in athletes. Rev Bras Med Esporte. (2020) 26(4):323–7. 10.1590/1517-869220202604214839

[51] Chrismas BC Taylor L Siegler JC Midgley AW. Muscle-damaging exercise 48 h prior to a maximal incremental exercise treadmill test reduces time to exhaustion: is it time to reconsider our pretest procedures? Res Sports Med. (2017) 25(1):11–25. 10.1080/15438627.2016.1258641 27855493

[52] Sieljacks P Degn R Hollaender K Wernbom M Vissing K. Non-failure blood flow restricted exercise induces similar muscle adaptations and less discomfort than failure protocols. Scand J Med Sci Sports. (2019) 29(3):336–47. 10.1111/sms.13346 30475424

[53] Shimomura Y Yamamoto Y Bajotto G Sato J Murakami T Shimomura N. Nutraceutical effects of branched-chain amino acids on skeletal muscle. J Nutr. (2006) 136(2):529S–32. 10.1093/jn/136.2.529S 16424141

[54] Stock MS Young JC Golding LA Kruskall LJ Tandy RD Conway-Klaassen JM. The effects of adding leucine to pre and post-exercise carbohydrate beverages on acute muscle recovery from resistance training. J Strength Cond Res. (2010) 24(8):2211–9. 10.1519/JSC.0b013e3181dc3a10 20634736

[55] Wernbom M Järrebring R Andreasson MA Augustsson J. Acute effects of blood flow restriction on muscle activity and endurance during fatiguing dynamic knee extensions at low load. J Strength Cond Res. (2009) 23(8):2389–95. 10.1519/JSC.0b013e3181bc1c2a 19826283

[56] Cardoso R Lumini-Oliveira JA Santos MJ Ramos B Matos LC Machado J. Acupuncture can be beneficial for exercise-induced muscle soreness: a randomised controlled trial. J Bodyw Mov Ther. (2020) 24(1):8–14. 10.1016/j.jbmt.2019.03.015 31987567

[57] Huang CC Lee MC Ho CS Hsu YJ Ho CC Kan NW. Protective and recovery effects of resveratrol supplementation on exercise performance and muscle damage following acute plyometric exercise. Nutrients. (2021) 13(9):3217. 10.3390/nu13093217 34579095 PMC8469037

[58] Váczi M Tékus É Kaj M Kőszegi T Ambrus M Tollár J. Changes in metabolic and muscle damage indicators following a single bout of jump training on stair versus at level. Acta Physiol Hung. (2013) 100(4):445–56. 10.1556/APhysiol.100.2013.010 24013940

[59] Huang C Wang Z Xu X Hu S Zhu R Chen X. Does acupuncture benefit delayed-onset muscle soreness after strenuous exercise? A systematic review and meta-analysis. Front Physiol. (2020) 11:666. 10.3389/fphys.2020.00666 32765287 PMC7379881

[60] Hill J Howatson G Van Someren K Leeder J Pedlar C. Compression garments and recovery from exercise-induced muscle damage: a meta-analysis. Br J Sports Med. Sept. (2014) 48(18):1340–6. 10.1136/bjsports-2013-092456 23757486

[61] Craig JA Bradley J Walsh DM Baxter GD Allen JM. Delayed onset muscle soreness: lack of effect of therapeutic ultrasound in humans. Arch Phys Med Rehabil. (1999) 80(3):318–23. 10.1016/S0003-9993(99)90144-2 10084441

[62] Page W Swan R Patterson SD. The effect of intermittent lower limb occlusion on recovery following exercise-induced muscle damage: a randomized controlled trial. J Sci Med Sport. (2017) 20(8):729–33. 10.1016/j.jsams.2016.11.015 28153608

[63] Connolly DA Sayers SP McHugh MP. Treatment and prevention of delayed onset muscle soreness. J Strength Cond Res. (2003) 17(1):197–208. 10.1519/00124278-200302000-00030 12580677

[64] Dakić M Toskić L Ilić V Đurić S Dopsaj M Šimenko J. The effects of massage therapy on sport and exercise performance: a systematic review. Sports. (2023) 11(6):110. 10.3390/sports11060110 PMC10302181 37368560

[65] Ammar A Bailey SJ Chtourou H Trabelsi K Turki M Hökelmann A. Effects of pomegranate supplementation on exercise performance and post-exercise recovery in healthy adults: a systematic review. Br J Nutr. (2018) 120(11):1201–16. 10.1017/S0007114518002696 30350760

[66] Cao H Li X Liu J. An updated review of the efficacy of cupping therapy. PLoS One. (2012) 7(2):e31793. 10.1371/journal.pone.0031793 22389674 PMC3289625

[67] Dudley GA Czerkawski J Meinrod A Gillis G Baldwin A Scarpone MM. Efficacy of naproxen sodium for exercise-induced dysfunction muscle injury and soreness. Clin J Sports Med. (1997) 7(1):3–10. 10.1097/00042752-199701000-00002 9117523

[68] Scottm H Jerryc D Jong D Brucer N Shirley R Philipg S. Effect of ibuprofen use on muscle soreness, damage, and performance: a preliminary investigation. Med Sci Sports Exerc. (1993) 25(1):9–17. 10.1249/00005768-199301000-00003 8423760

[69] Donnelly A McCormick K Maughan R Whiting P Clarkson PM. Effects of a non-steroidal anti-inflammatory drug on delayed onset muscle soreness and indices of damage. Br J Sports Med. (1988) 22(1):35–8. 10.1136/bjsm.22.1.35 3370401 PMC1478505

[70] Kuipers H Keizer H Verstappen F Costill DJ. Influence of a prostaglandin-inhibiting drug on muscle soreness after eccentric work. Int J Sports Med. (1985) 6(6):336–9. 10.1055/s-2008-1025866 4077362

[71] Kitsuksan T Nartprayut S Sawangmanee P Klainin W Laddawong T Boonkerd C. The application of tissue flossing during plyometric exercise for the prevention of delayed onset muscle soreness. Sci Technol Asia. (2021) 26:145–54.

[72] Meixner CR Nagel AM Höger SA Gast LV Wiesmueller M Uder M. Muscle perfusion and the effect of compression garments in delayed-onset muscle soreness assessed with arterial spin labeling magnetic resonance imaging. Quant Imaging Med Surg. (2022) 12(9):4462–73. 10.21037/qims-21-1104 36060583 PMC9403597

[73] Fleckenstein J Friton M Himmelreich H Banzer W. Effect of a single administration of focused extracorporeal shock wave in the relief of delayed-onset muscle soreness: results of a partially-blinded randomized controlled trial. Arch Phys Med Rehabil. (2017) 98(5):923–30. 10.1016/j.apmr.2016.11.013 27993588

[74] Elcadi GH Andersson L Hemmingsson P. The impact of eccentric resistance training on muscle proprioception and movement control. Eur J Appl Physiol. (2023) 123(4):789–802.

[75] Wiecha S Posadzki P Prill R Płaszewski M. Physical therapies for delayed onset muscle soreness: a protocol for an umbrella and mapping systematic review with meta-meta-analysis. J Clin Med. (2024) 13(7):2006. 10.3390/jcm13072006 38610771 PMC11012564

[76] Fleck C Posadzki P Duhem S. The effect of neurodynamic mobilization and foam rolling on delayed onset muscle soreness. J Manual Ther. (2021) 26(3):243–51.

[77] Wiewelhove T Döweling A Schneider C Hottenrott L Meyer T Kellmann M. A meta-analysis of the effects of foam rolling on performance and recovery. Front Physiol. (2019) 10:376. 10.3389/fphys.2019.00376 31024339 PMC6465761

[78] MacDonald GZ Penney MDH Mullaley ME Cuconato AL Drake CDJ Behm DG. An acute bout of self-myofascial release increases range of motion without a subsequent decrease in muscle activation or force. J Strength Cond Res. (2013) 27(3):812–21. 10.1519/JSC.0b013e31825c2bc1 22580977

[79] Romero-Moraleda B La Touche R Lerma-Lara S Ferrer-Peña R Paredes V Peinado AB. Neurodynamic mobilization and foam rolling improved delayed-onset muscle soreness in a healthy adult population: a randomized controlled clinical trial. PeerJ. (2017) 5:e3908. 10.7717/peerj.3908 29043110 PMC5642244

[80] Loenneke JP Wilson JM Wilson GJ. Blood flow restriction: implications for delayed onset muscle soreness. Sports Med. (2023) 53(2):145–60.

[81] Hughes L Paton B Rosenblatt B Gissane C Patterson SD. Blood flow restriction training in clinical musculoskeletal rehabilitation: a systematic review and meta-analysis. Br J Sports Med. (2017) 51(13):1003–11. 10.1136/bjsports-2016-097071 28259850

[82] Wilson JM Loenneke JP Jo E. The role of blood flow restriction in muscle recovery: a comprehensive review. Strength Cond J. (2022) 44(1):48–62.

[83] Chi LM Lin LM Chen CL Wang SF Lai HL Peng TC. The effectiveness of cupping therapy on relieving chronic neck and shoulder pain: a randomized controlled trial. Evid Based Complement Alternat Med. (2016) 2016:7358918. 10.1155/2016/7358918 27073404 PMC4814666

[84] Sluka KA Bjordal JM Marchand S Rakel BA. What makes transcutaneous electrical nerve stimulation work? Making sense of the mixed results in the clinical literature. Phys Ther. (2013) 93(10):1397–402. 10.2522/ptj.20120281 23641031 PMC3788712

[85] Halim HK Oliveira CA Mattiello SM. The role of NMES in exercise-induced muscle soreness recovery. Clin Biomech. (2022) 92:105607.

[86] Leal-Junior ECP Vanin AA Miranda EF De Carvalho PTC Dal Corso S Bjordal JM. Effect of phototherapy (low-level laser therapy and light-emitting diode therapy) on exercise performance and markers of exercise recovery: a systematic review with meta-analysis. Lasers Med Sci. (2015) 30(2):925–39. 10.1007/s10103-013-1465-4 24249354

[87] Baroni BM Leal Junior ECP Pinto RS. Effect of heat therapy on muscle recovery after resistance exercise. J Sports Sci Med. (2010) 9(3):437–43.

[88] Bleakley CM Davison GW Eston RG. Cryotherapy and inflammation: evidence beyond the cardinal signs. Phys Ther Sport. (2012) 13(3):170–5. 10.1179/1743288X10Y.000000001422814451

[89] Versey NG Halson SL Dawson BT. Effect of contrast water therapy duration on recovery of running performance. Int J Sports Physiol Perform. (2012) 7(2):130–40. 10.1123/ijspp.7.2.130 22173197

[90] Lee JH Choi TY Lee MS Lee H Shin BC Lee H. Acupuncture for acute low back pain: a systematic review. Clin J Pain. (2013) 29(2):172–85. 10.1097/AJP.0b013e31824909f9 23269281

[91] Dommerholt J Fernández-de-las-Peñas C Gerwin RD. Trigger Point Dry Needling: An Evidence and Clinical-Based Approach. 2nd ed. Elsevier (2018). ISBN: 978-0-7020-7416-5.

[92] Fatouros I Jamurtas T Leontsini D Taxildaris K Aggeloussis N Kostopoulos N. Evaluation of plyometric exercise training, weight training, and their combination on vertical jumping performance and leg strength. J Strength Cond Res. (2000) 14(4):470–6. 10.1519/00124278-200011000-00016

[93] Markovic G. Does plyometric training improve vertical jump height? A meta-analytical review. Br J Sports Med. (2007) 41(6):349–55. 10.1136/bjsm.2007.035113 17347316 PMC2465309

[94] Proske U Morgan DL. Muscle damage from eccentric exercise: mechanism, mechanical signs, adaptation, and clinical applications. J Physiol. (2001) 537(2):333–45. 10.1111/j.1469-7793.2001.00333.x 11731568 PMC2278966

[95] Viitasalo JT Hämäläinen K Mononen HV Salo A Lahtinen J. Biomechanical effects of fatigue during continuous hurdle jumping. J Sports Sci. (1993) 11(6):503–9. 10.1080/0264041930873002 8114175

[96] Markovic G Mikulic P. Neuro-musculoskeletal and performance adaptations to lower-extremity plyometric training. Sports Med. (2010) 40(10):859–95. 10.2165/11318370-000000000-00000 20836583

[97] Häkkinen K Komi PV. Changes in neuromuscular performance in voluntary and reflex contraction during strength training in man. Int J Sports Med. (1983) 4(4):282–8. 10.1055/s-2008-1026051 6228532

[98] Hunt JEA Coelho MOC Buxton S Butcher R Foran D Rowland D. Consumption of New Zealand blackcurrant extract improves recovery from exercise-induced muscle damage in non-resistance trained men and women: a double-blind randomised trial. Nutrients. (2021) 13(8):2875. 10.3390/nu13082875 34445035 PMC8399782

[99] Taube W Leukel C Lauber B Gollhofer A. The drop height determines neuromuscular adaptations and changes in jump performance in stretch-shortening cycle training. Scand J Med Sci Sports. (2012) 22(5):671-83. 10.1111/j.1600-0838.2011.01293.x 21457355

[100] Eston R Peters D. Effects of cold water immersion on the symptoms of exercise-induced muscle damage. J Sports Sci. (1999) 17(3):231-8. 10.1080/026404199366136 10362390

[101] Kay AD Rubley B Talbot C Mina M Baross AW Blazevich AJ. Stretch imposed on active muscle elicits positive adaptations in strain risk factors and exercise-induced muscle damage. Scand J Med Sci Sports. (2018) 28(11):2299–309. 10.1111/sms.13251 29943872

[102] Cao W Qiu J Cai T Yi L Benardot D Zou M. Effect of D-ribose supplementation on delayed onset muscle soreness induced by plyometric exercise in college students. J Int Soc Sports Nutr. (2020) 17(1). 10.1186/s12970-020-00371-8 PMC7418385 32778175

[103] Harrison DC Doma K Rush C Connor J. Acute effects of exercise-induced muscle damage on sprint and change of direction performance: a systematic review and meta-analysis. Biol Sport. (2024) 41(3):153–68. 10.5114/biolsport.2024.131823 38952917 PMC11167466

[104] De França IM Cerqueira MS Barros de Sousa Oliveira YM Patterson SD de Brito Vieira WH. Effects of ischemic pre and postconditioning on indirect markers of muscle damage: a systematic review and meta-analysis. Muscles Ligaments Tendons J. (2024) 14(1).

[105] Wilke J Behringer M. Is “delayed onset muscle soreness” a false friend? The potential implication of the fascial connective tissue in post-exercise discomfort. Int J Mol Sci. (2021) 22(17):9482. 10.3390/ijms22179482 34502387 PMC8431437

